# Accumulation of *Dinophysis* Toxins in Bivalve Molluscs

**DOI:** 10.3390/toxins10110453

**Published:** 2018-11-02

**Authors:** Juan Blanco

**Affiliations:** Centro de Investigacións Mariñas, Pedras de Corón s/n, 36620 Vilanova de Arousa, Spain; juan.carlos.blanco.perez@xunta.gal; Tel.: +34-886-206-340

**Keywords:** okadaic acid, pectenotoxins, *Dinophysis* toxins, accumulation, digestion, biotransformation, compartmentalization, depuration, kinetics

## Abstract

Several species of the dinoflagellate genus *Dinophysis* produce toxins that accumulate in bivalves when they feed on populations of these organisms. The accumulated toxins can lead to intoxication in consumers of the affected bivalves. The risk of intoxication depends on the amount and toxic power of accumulated toxins. In this review, current knowledge on the main processes involved in toxin accumulation were compiled, including the mechanisms and regulation of toxin acquisition, digestion, biotransformation, compartmentalization, and toxin depuration. Finally, accumulation kinetics, some models to describe it, and some implications were also considered.

## 1. Introduction

### 1.1. Dinophysis-Produced Toxins

**Figure 1:**
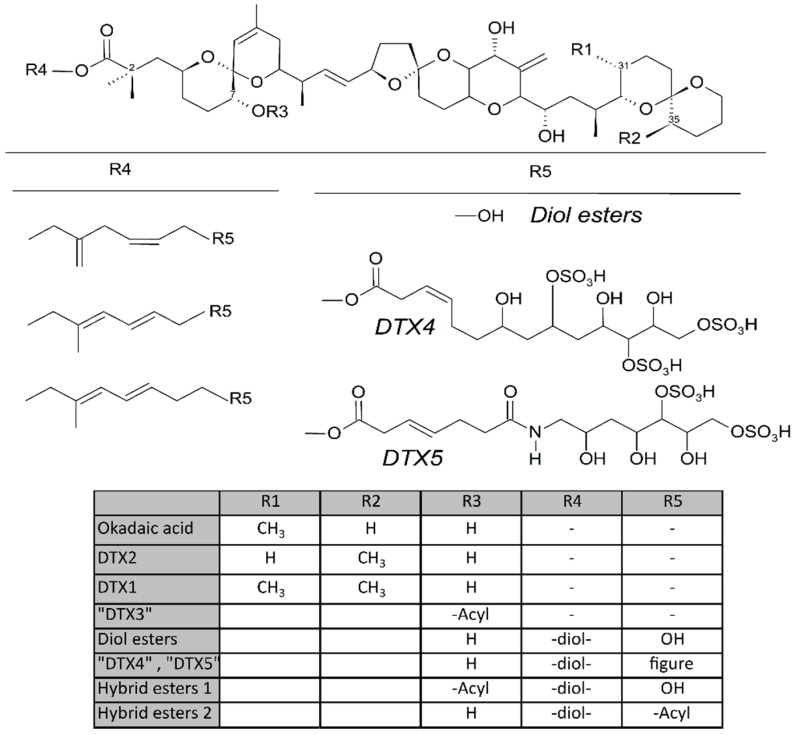
Structure of the toxins of the okadaic acid group. R4 and R5 are some examples of structures which may be more complex.

Some dinoflagellates of the genus *Dinophysis* produce toxins belonging to the okadaic acid group (okadaic acid and dinophysistoxins, OA and DTXs, respectively) and/or to pectenotoxins (PTXs). Both groups of toxins are polyethers having a linear structure in the OA group and a macrocyclic lactone in pectenotoxins. The compounds in the OA group have a terminal carboxylic function, which in some cases, esterifies diols or other compounds, and a hydroxyl in C-7 that is frequently esterified with fatty acids to yield the group of derivatives generically known as “DTX3” (Figure 1). The macrolactone cycle of PTXs could be opened to produce seco-acids that in turn can be esterified with fatty acids (Figure 7).

Dinophysistoxin-1 (DTX1) was identified in 1982 as the substance responsible for a toxic syndrome (Diarrhetic Shellfish Poisoning, DSP) [1,2], which affected more than 1600 people in Japan [3]. This toxin is a 35-R-Methyl derivative of okadaic acid, a compound that had previously been isolated from two sponges of the genus *Halichondria* (*H. okadai* and *H. melanodocia*) [4], and which since then, has been associated with numerous DSP outbreaks occurring all over the world [5,6,7,8,9]. The allowable levels in shellfish of this toxin and other toxins or derivatives of the same group have been regulated in many countries. In Europe and other areas, the established regulatory threshold is 160 µg OA-eq/Kg of edible product (quantified together with pectenotoxins) [10,11,12].

Pectenotoxins have never been linked to any human intoxication [13], but they were discovered because they co-elute with the toxins of the okadaic group and are lethal to mice by intraperitoneal injection in the bioassays typically used to monitor DSP toxins. The regulatory level in Europe is the same as the one for the toxins of the OA group (quantified jointly) [11].

The threat that these toxins pose to human health makes it mandatory to implement monitoring systems. Legal/regulatory strategies must allow for the proper management of marine resources (including aquaculture) to preserve public health and minimize the economic losses of fishermen and farmers [14]. Both monitoring and management have costs and these can be high, depending on the importance and value of the resources and means of commercialization [15].

Bivalves, retain, ingest, and digest *Dinophysis* cells, bioaccumulate the toxins they contain and biotransform them into derivatives that could have different toxicities than their parent toxins. Understanding these processes is essential to developing predictive capability of the intensity and duration of toxic episodes of *Dinophysis*, and consequently, using the abundance of its populations as a warning in monitoring systems and designing mechanisms to mitigate their impact by means of the acceleration of the depuration process or the reduction of toxin uptake.

### 1.2. Toxins in Phytoplankton

Different species (or strains) of *Dinophysis* produce different toxins. Phytoplanktonic populations usually contain free toxins (the main toxin structure without esterifying or being esterified with any other compound). Thus, okadaic acid, DTX1, DTX2, and PTX2 (PTX11 and 12 to a lesser extent) are the main toxins found.

Okadaic acid is present in many *Dinophysis* species and is dominant in European waters. In Japan, Chile, and the U.S., DTX1 appeared more frequently [16,17,18,19]. DTX2 seems to be practically restricted (with the exception of a few samples from Baja California, Mexico [20]) to the Atlantic coast of Europe (Ireland [21] (where it was first identified), Spain [22,23], Portugal [24], Southern Norway [25], Great Britain [26]), Northern Africa (Morocco [27], Tunisia [28]), and the Mediterranean Sea [29],) and is mostly (or exclusively) associated with *Dinophysis acuta* [21,30,31].

Derivatives of the free toxins in which the carboxylic function esterifies diols (diol esters), triols, or more complex molecules (DTX4, DTX5) (Figure 1) have been described from certain OA-producer *Prorocentrum* species [32,33,34,35,36,37,38,39]. In some species of the genus *Dinophysis*, such as *D. acuta* [40,41,42], diol esters have been found and their presence is suspected in others like *D. ovum* and *D. acuminata*, in view of the increase in free OA produced by the alkaline hydrolysis of the extracts of a bloom [43,44]. Some other species, such as *D. fortii* [40], and probably some strains of the cited ones, do not seem to contain these kinds of compounds.

Pectenotoxins—mainly PTX2, but sometimes also PTX11 and 12 [42,45,46]—are produced by several *Dinophysis* species [45,47,48,49,50,51,52,53,54,55,56,57,58,59,60]. Frequently their detection is concurrent with that of toxins of the OA group, but in some cases, only pectenotoxins seem to be produced [17,61,62].

## 2. Ingestion

Toxins can be acquired by bivalves in two ways: (1) Directly from the dissolved phase, and (2) from the cells or particulate matter that contain them.

The uptake of okadaic acid by bivalves from the dissolved phase has been demonstrated [63,64]. The capability of this toxin to pass through lipid bilayers by forming aggregates [65] or dimers [66] had been previously shown. Other lipophilic toxins—the azaspiracids—for which this capability has not been demonstrated, could also be acquired by mussels in a similar way [67]. Li et al. [63], observed that the concentration of OA in bivalves exposed to dissolved toxin exceeded the levels that could be expected in view of concentrations in the water, suggesting that an active uptake mechanism could exist, at least in the digestive gland.

**Figure 2:**
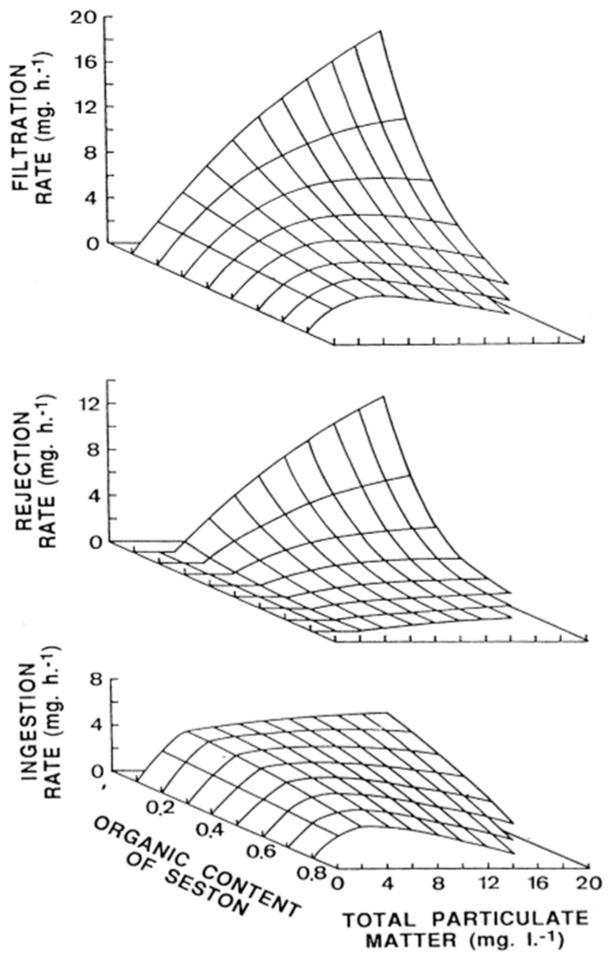
Filtration, ingestion, and rejection of seston by the cockle *Cerastoderma edule* as a function of organic content and seston concentration. Reproduced with permission from Iglesias et al. [75], published by Elsevier 1996.

Most toxins, notwithstanding, are retained by bivalves together with the cells that produce them. Bivalves pump water, with the particles suspended in it, through the gill and retain them in a proportion which depends, among other factors, on their size. The feeding process comprises water pumping and filtration, pre-ingestive particle selection, ingestion, post-ingestive selection, and food amount regulation (reviewed in Dame [68] and Gosling [69]). Most bivalves retain particles larger that 5–7 µm with efficiencies of 100% [70,71,72,73], and consequently, can retain *Dinophysis* cells (mostly between 45 and 85 µm [74]) with high efficiency. Therefore, practically all particles pumped through the gill are filtered and retained. The pumping rate depends mostly on the species, size of the individuals, and concentration, as well as the quality of the particles suspended in water (seston). In general, filtration is low at low seston concentrations, maximum at intermediate levels, and submaximal when the concentration is high (reviewed in Gosling [69]), as is the case, for example, of the cockle *Cerastoderma edule* in most conditions [75] (Figure 2).

When the filtration efficiency is close to 100%, the filtration rate and clearance rate (the rate at which particles are withdrawn from water) are equivalent.

Filtration rate is a species-specific characteristic that can explain, at least in part, the differences in the accumulation of *Dinophysis*-produced toxins between species. Oysters, for example, accumulate fewer toxins than mussels [76,77,78,79], and their maximum filtration rate is, in general, lower [80,81]. Filtration rate is dependent on the gill area, and consequently, proportional to (approximately) the square of the body length (L^2^), and also approximately to body weight (W^2/3^) (reviewed by Cranford et al. [82] and Gossling [69], which means that smaller individuals of the same species filtrate more cells or particles in relation to their body weight than larger ones.

The phytoplankton species may also affect filtration and clearance rates, as has been demonstrated for some PSP producing species of *Alexandrium* [83] and some *Pseudo-nitzschia* (whether or not they produce domoic acid) [84], but there is no evidence of these kinds of effects being caused by any natural population of *Dinophysis*. Two species of scallops, *Patinopecten yessoensis* and *Mimachlamys nobilis*, were shown to be affected by cultures of a PTX2-producer, *Dinophysis acuta*, but to a degree that was not dependent on the toxin quota of the cultured cells [85]. Another okadaic acid-producing species, *Prorocentrum lima*, has been shown to reduce the filtration rate of mussels [86], and recently, Li et al. [63] also found a reduced clearance rate in mussels exposed to okadaic acid isolated from the same species. In none of these cases can the possible contribution of other biologically active substances be ruled out.

In some cases, a proportion of the cleared particles is rejected, in a degree that is dependent, at the very least, on the seston concentration and on its organic content, with maximum rejection levels at high concentrations of inorganic particulate matter. *Dinophysis* blooms are associated with a wide range of particulate matter concentrations, ranging from very low (in cases where hardly any other phytoplankton species are present) to very high (in cases where *Dinophysis* are only minor accompanying species). Therefore, a constant response of bivalves, in terms of rejection, would not be expected. Sampayo et al. [87] and Haamer [88] found that the toxicity degree of bivalves exposed to *Dinophysis* populations was lower when the abundance of accompanying species was low, which, among other causes, could be due to increased rejection under these conditions.

Not all particles that are retained by the gill are ingested afterwards. Some particles are negatively selected and rejected through the production of pseudofaeces [75,89,90,91,92] or other mechanisms [93,94]. In general, particles with a high organic content, which include phytoplankton, and therefore *Dinophysis* cells, are preferentially ingested [90]. Size may also play an important role in particle selection. Inorganic particles, with the same shape, larger than the threshold size (depending on the species) were found to be rejected preferentially [95]. Mafra et al. [84] also found that the oyster *Crassostrea virginica* preferentially rejects large cells of *Pseudo-nitzschia multiseries*—a diatom producer of the ASP toxin domoic acid—an occurrence which they associated with the fact that the large cells exceeded the width of the principal filament aperture (approx. 68 µm). Even when *Dinophysis* cells are above that size threshold, there is no evidence that they (or other organic particles) are rejected preferentially. Contrarily, it seems that the mussel *Mytilus galloprovincialis* can ingest *Dinophysis* cells preferentially over other phytoplanktonic species, in view of the gut remains [96].

Intraspecific differences in OA accumulation during the early stages of a *Dinophysis* bloom (therefore, probably related to cell ingestion) have also been found in mussels. These differences would have a genetic basis, as a heritability greater than 30% was estimated [97].

## 3. Post-Ingestive Selection and Regulation of Food Processing

**Figure 3:**
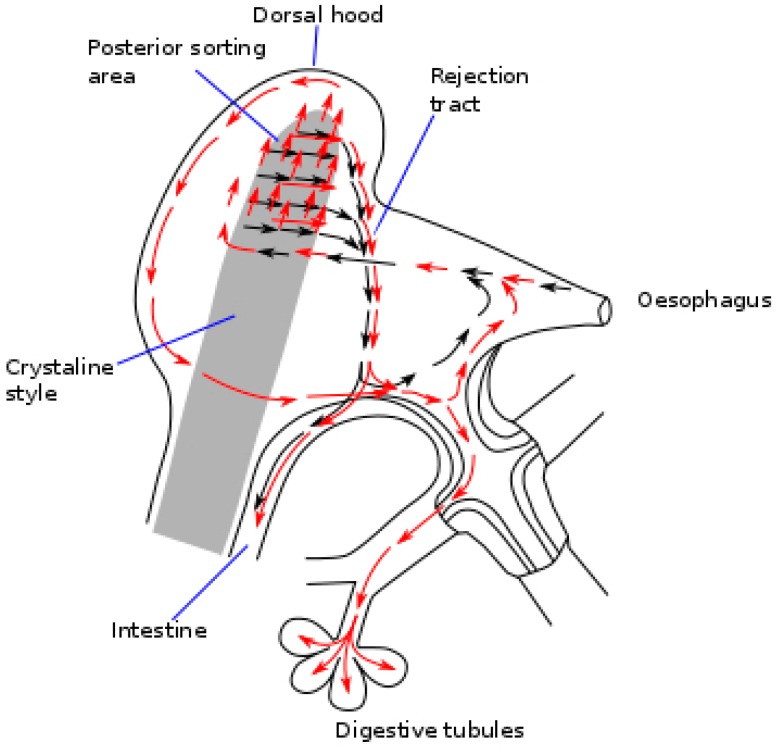
Schematic representation of the particle flow through the digestive system of a bivalve (redrawn and simplified from Owen [98]). Black arrows represent large particles and red arrows small particles.

Once ingested, the *Dinophysis* cells, jointly with other particles go through the esophagus to the stomach and the crystalline stylus sac where they are broken down [98] into fine fragments, before being transferred to the digestive tubules. The walls of the stomach of the bivalves have a complex network of ciliated folds that are believed to act by sorting particles [99]. If the number of particles ingested is low, a high proportion of the large particles are recurrently sent to the crystalline style sac for additional processing, and another proportion is directly rejected and sent to the intestine. If the amount of food ingested is high, a larger proportion of the ingested cells is diverted unprocessed to the intestine and eliminated with feces (Figure 3). The higher the volume of ingested material, the shorter the time it will stay in the digestive system (gut passage time, GPT), and it will consequently go through less processing and digestion, leading to a lower absorption efficiency of the organic matter [100,101]—including the toxins [102,103]—it contains. These processes could help explain why the bivalves exposed to *Dinophysis* populations acquire less toxicity when the accompanying populations of other phytoplankton species are abundant [87,88].

The ingested particles could be selectively diverted to the intestine and eliminated with feces without further processing by post-ingestive selection, which has been documented for several species and types of particles [92,104,105,106]. This kind of selection has not been demonstrated for *Dinophysis*, but it was shown for another okadaic acid producer [107,108], *Prorocentrum lima*, where this mechanism was hypothesized as being a way to reduce the accumulation of the toxin and avoid its possible effects [108].

## 4. Digestion and Uptake

**Figure 4:**
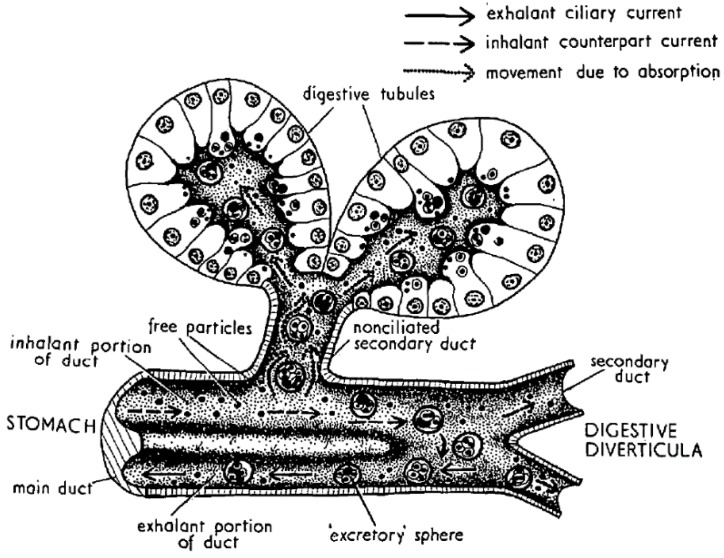
Structure of the digestive tubules and diverticula, showing incoming particles and outgoing excretory spheres (rejection bodies). Reproduced with permission from Owen [98], published by Company of Biologists 1955.

Digestion in bivalves has two components: one is extracellular, which takes place mostly in the stomach and crystalline style sac, and the other is intracellular, which takes place mostly in the cells of the digestive tubules (Figure 4). In the first step, the ingested particles, including *Dinophysis* and other phytoplankton cells, are broken down and consequently, the released substances are subjected to the action of both the autolytic enzymes of the ingested phytoplankton and the digestive enzymes secreted by the bivalve. The pH of the digestive system is also different from the one in seawater but it is not extreme, with its minimum value being around 6. Neither the enzymatic activity nor the pH levels seem to degrade or transform the main *Dinophysis*-produced toxins [64,109], as the amounts of toxin ingested and excreted have been found to be approximately the same. At least some of their derivatives, notwithstanding, could be hydrolyzed to the main toxins or to other (simpler) derivatives. The enzymes of the diatom *Thalassiosira weissflogii*, for example, were shown to quickly convert DTX4 and DTX5 to diol-esters, and those, at a lower rate, to the main toxins [110]. The same processes are known to take place with autolytic enzymes of the OA producer *Prorocentrum lima*, as shown by the fact that the cell concentrates must be boiled to inactivate enzymes to obtain these compounds [36,111,112]. It can be expected that enzymes of this kind will be released into the stomach after cell breakage and catalyze the hydrolysis as discussed earlier. The digestive enzymes of the bivalves also play a role in transformations of this type as these compounds are quickly hydrolyzed by esterases [34] and bivalves secrete enzymes of this group [69,113]. Some studies of the time-course of OA, DTX2 (and their conjugated forms) depuration in the mussel *Mytilus galloprovincialis* showed a quick decrease in conjugated forms just after *Dinophysis* ingestion stopped, which was interpreted as corresponding to the hydrolysis of the conjugated forms acquired from the *Dinophysis* cells [44,114]. Mackenzie et al. [115] isolated a digestive esterase from the green mussel *Perna canaliculus*, which has the capability of hydrolyzing diol-esters, as well as 7-O-acyl esters, of OA (which may also be also present in seston after OA is biotransformed by *Dinophysis* consumers). It is possible that not all diol-esters are hydrolyzed at the same rate, as the activity of the enzyme varies noticeably with the chain length of several 4-nitrophenyl esters tested [115].

Much less information is available on pectenotoxins. The main change induced by digestive processes is the opening of the macrolactone ring to produce a secoic acid. As discussed earlier, the esterase isolated from *P. viridis* catalyzes this transformation of some, but not all, pectenotoxins. PTX2 and PTX1, for example, are readily hydrolyzed while PTX11, PTX2c, and other analogues are not affected. Additionally, at least the latter two compounds act as competitive inhibitors of PTX2 hydrolysis [115], which may also be true of equivalent enzymes of other bivalve species.

From the stomach, the partially digested material is diverted to the digestive tubules where extracellular digestion is completed, and where intracellular digestion takes place [69,113]. The uptake of the toxins of the OA group by digestive cells has been the object of very few studies, but there are several mechanisms that could be involved. Rossignoli [64] found that OA was taken up by fragments of digestive gland much faster when supplied in dissolved form than in an emulsion of oil droplets. Moreover, it was recently found that dissolved OA and DTX1 can be taken out by different tissues of the mussel *Mytilus galloprovincialis* [63], but especially by the digestive gland. Even when, at the pH levels found in the digestive gland, the main toxins of the OA group are ionized, in view of their pKa [116], they were shown to be able to self-assemble [66] or to form aggregates of several molecules [65] in a way that hides the charged parts of the molecule, allowing them to pass through the lipid bilayers (as cell membranes). Diol-esters are less polar than their corresponding main toxins and the carboxylic function, being combined with a diol, cannot be charged. Hence, they could be taken up more easily than the free toxins. It would be expected that the toxins taken up by this mechanism are initially stored in the cytosol, as was found for OA by Rossignoli and Blanco [117] and by Guéguen et al. (mostly) [118].

**Figure 5:**
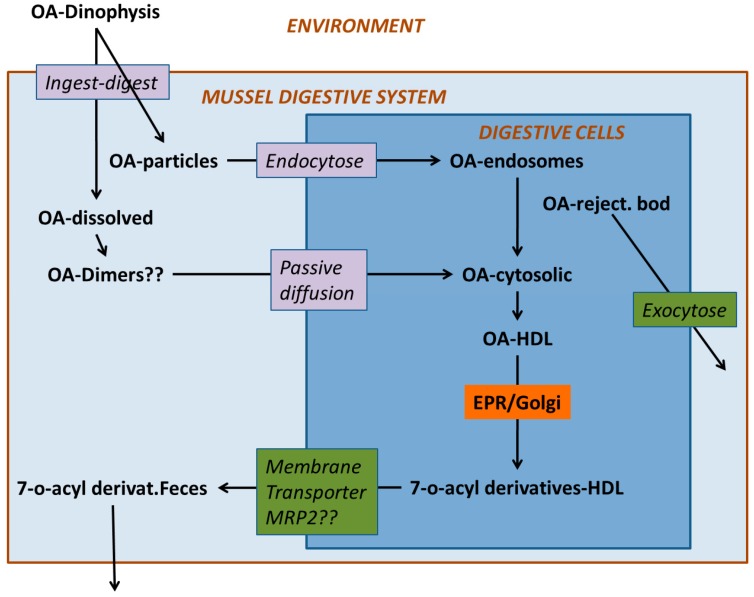
Hypothetical steps involved in the accumulation of toxins in the okadaic acid (OA) group.

Some endocytic mechanisms, such as phagocytosis or pinocytosis that are involved in the uptake of different components of food by digestive cells, do not require the toxins to be uncharged because they do not need to pass through the cellular membrane (Figure 5). Dissolved OA could be absorbed mainly by means of phagocytosis when associated with debris of *Dinophysis* cells, or by pinocytosis, in addition to diffusion through the membrane, when it is in solution. In both cases, the toxins would enter the cell inside endosomes that would be progressively converted into lysosomes. Guéguen et al. [118] found a noticeable proportion of the cellular okadaic acid to be located in lysosomes, which means that this route of uptake could also be important. Phagocytosis has been suggested for highly lipophilic xenobiotics [119], with an octanol-water partition coefficient ≥ 4 (log P), because they are mostly associated (adsorbed or dissolved into them) with organic particles or lipid droplets [120].

Phytoplanktonic pectenotoxins, which have a polarity similar to that of OA, and which are not ionized at a pH below 8, are expected to share the same uptake routes.

The dominance of one route or another is probably a complex mixture of the distribution of toxins in the lumen of the digestive tubules, the concentration of free toxins in the cytosol of the cells and the rates of diffusion (passive or facilitated) or phagocytosis.

## 5. Compartmentalization

When the toxins enter the digestive gland cells, they are distributed heterogeneously between the different types and the different parts of the cells. Okadaic acid was shown to be stored preferentially in the digestive cells of *M. galloprovincialis* rather than the secretory cells [121] (the two main cellular types that integrate the digestive tubules) [113,122]. The structure and function of each cellular type could explain this preferential storage, as secretory cells present less surface to tubule lumen than digestive cells, and their function as secretory cells does not include endocytic processes.

Once inside the cells, okadaic is mostly located in the cytosol and bounded to (or dissolved into) high-density lipoprotein(s) (HDL) [117] (the same was observed for acyl-derivatives of OA, unpublished information), but in some cases, a noticeable proportion of OA could be also found in lysosomes or similar cellular structures [118]. It seems very likely that the toxin contained in the lysosomes entered the cell by means of an endocytic mechanism and in the cytosolic fraction it entered the cell in dissolved form and/or was transferred to the cytosol from the lysosomes.

The association with HDLs probably has a transport function, since this group of proteins is strongly linked to the transport of lipophilic substances in the organism. In humans, for example, HDLs in association with ABC membrane transporters are responsible for removing excess cholesterol from cells and transporting it to the liver and other steroidogenic tissues to be metabolized and excreted [123].

There is no information available on the cellular or subcellular distribution of pectenotoxins, but it would seem likely that they share this with other compounds of similar polarity, such as OA or cholesterol.

Anatomically, both pectenotoxins and toxins of the OA group are heterogeneously distributed among organs and/or tissues. Okadaic acid has been shown to be concentrated especially in the digestive gland of the mussels *Mytilus galloprovincialis* [124], *M. edulis* [86,125,126,127], and in the scallop *Argopecten irradians* [108]. It also appears to be the case with two Australian scallop species, *Pecten fumatus* [128] and *P. maximus* [129], the clams *Spisula solida* and *Donax trunculus* [130], and the razor clam *Pinna bicolor* [128]. Notwithstanding, recently in *Crenomytilus grayanus*, it was found that the acyl-esters of OA were more abundant in other organs than in the digestive gland (quantified by means of ELISA assay) [131].

Little information is available on pectenotoxins, even though it is generally admitted that the digestive gland is the main accumulator of this group of toxins, and this organ was used to isolate pectenotoxins as a step prior to their purification (e.g., Daiguji et al. [48]). In the Chilean surf clam *Mesodesma donacium* PTX2 and PTX2sa were 10-fold more concentrated in the digestive gland than in the remaining tissues, and the esters were nearly absent outside the digestive gland (approx. 300 times less concentrated) [132]

It is highly likely that compartmentalization influences depuration. As the main organs involved in excretion in bivalves are the kidney and digestive gland, the toxins located in other body tissues would probably be transported to these organs before starting depuration, which would slow down the process.

## 6. Transformation

**Figure 6:**
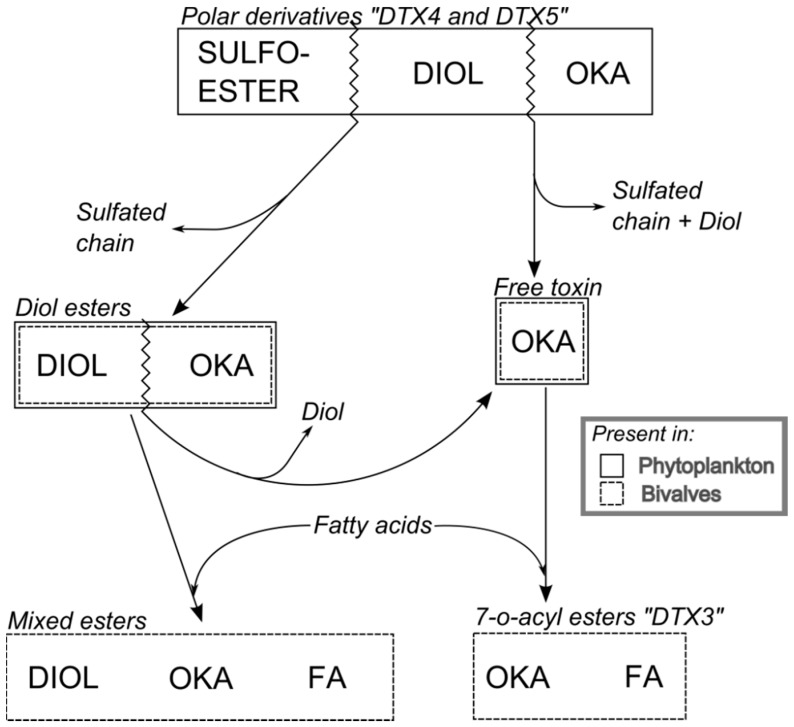
Main transformations of the toxins of the okadaic acid group. Labels inside the boxes indicate the moieties that constitute the molecule. Zigzag lines indicate the bonds that are broken to generate other compounds. The line(s) of each box indicate whether the compounds are found in phytoplankton or in bivalves. From Reguera et al. [74].

**Figure 7:**
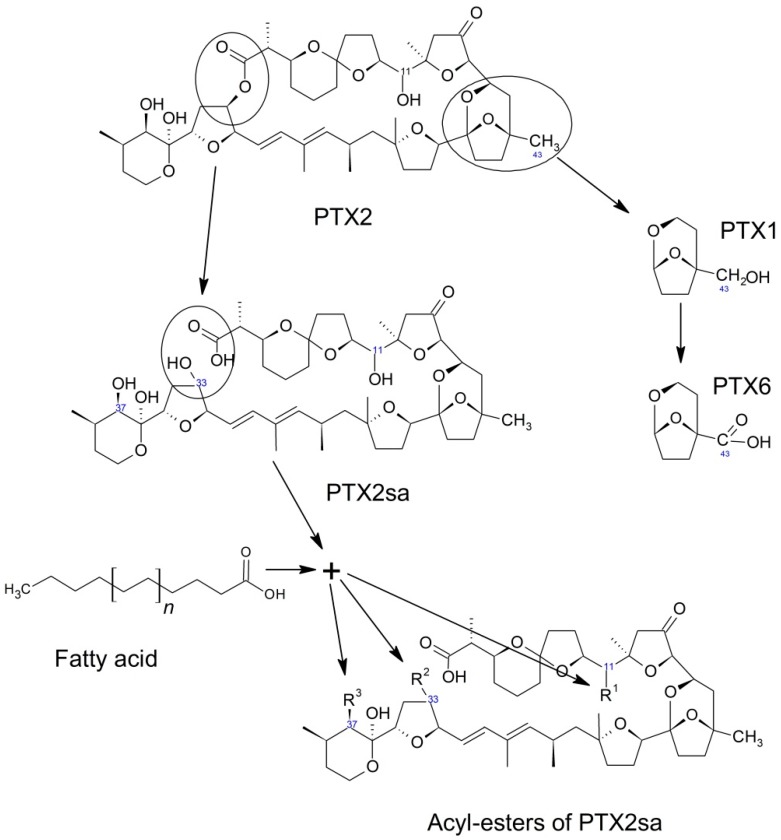
Transformations of PTX2 in bivalves.

The toxins produced by *Dinophysis* undergo transformations during the extracellular digestive process, as is the case of the hydrolysis of conjugated forms of the toxins in the OA group (Figure 6), and the formation of secoic acids of the pectenotoxins (Figure 7). Thereafter, they are partially transformed inside the cells of the bivalves. The main transformation route is the esterification with fatty acids of different chain lengths. In the toxins of the OA group, the hydroxyl group in C7 is frequently esterified with fatty acids, forming 7-O-acyl derivatives, generically known as DTX3 [133].

The free toxins undergo an esterification with fatty acids [134,135], where the microsomal fraction of the digestive gland cells—probably originating from the endoplasmic reticulum—is involved [134]. In some cases it seems that diol- or triol-esters which may not be partially hydrolyzed during the digestion process, are also esterified yielding mixed (or hybrid) esters, where the carboxylic function of the OA (or other analogues) is esterifying a diol or triol and a hydroxyl (in C7 of the OA or in any location of the diol/triol) is esterified with a fatty acid [38]. The proportion of the different fatty acids involved in the formation of the esters is variable, depending mainly on the bivalve species. Linear fatty acids with an even number of carbon atoms are frequent in all molluscs [136,137,138,139,140,141], and the esters of OA and PTX are usually formed with these fatty acids. Nevertheless, some infaunal species like cockles and clams, seem to have a noticeable proportion of odd-chain and branched-chain fatty acids involved in the esterification of the toxins [136], which Vale [142] hypothesized as possibly being caused by bacterial action.

Not all bivalve species have the same esterification capability and not all toxins are esterified at the same rate. In most of the studied bivalve species, the esterification is fast, reaching nearly 100% soon after the supply of free toxins is interrupted. This is the case, for example, of the cockle *Cerastoderma edule*, the peppery furrow shell *Scrobicularia plana*, the carpet shell *Venerupis pullastra*, the Pacific oyster *Crassostrea japonica* (*Magallana gigas*), the razor clams *Ensis* spp., *Ruditapes decussatus* [143,144], the European flat oyster *Ostrea edulis* [26,144,145], the surf clam *Spisula* [146], the littleneck clam *Leukoma staminea* [143], the scallop *Patinopecten yessoensis* [147,148], the Manila clam *Ruditapes philippinarum* (unpublished data), and some Chilean mussels (the blue mussel *M. chilensis* and the ribbed mussel *Aulacomya ater* [149]). In other mussels, such as *Mytilus edulis* [144], *M. galloprovincialis* [44,114,130,134,148,150,151,152], *M. coruscus* [139], and *Crenomytilus grayanus* [131]), as well as in some other species, like the clam *Donax trunculus* [136] or the variegated scallop *Aequipecten opercularis* (unpublished observation), the proportion of esterified toxins is usually much lower than 100%.

Different toxins could be differentially esterified, depending on the species. In general, it seems that the species that readily esterify OA (most infaunal species and oysters) do not show important differences between toxins, whilst other species, such as *Mytilus galloprovincialis* or *M. edulis*, for example, where OA is only partially esterified, esterify other toxins of the same group much less efficiently. In Norway [144], the flat oyster *Ostrea edulis*, was shown to contain a high proportion of esterified OA (86%) and also high proportions of esterified DTX1 and DTX2 (93 and 83%, respectively), whilst the blue mussel *Mytilus edulis*, with only 41% of the OA esterified, contained substantially lower proportions of esterified DTX1 and DTX2 (27 and 21%, respectively). The same pattern was found in an intoxication experiment with the same species and toxins involved [145], and in the early work of Marr et al. [141]. In Portugal, Vale and Sampayo [150] found no difference in the esterification of OA and DTX2, in most clams, cockle, and oyster, which esterified these compounds almost completely. However, they did find a substantially lower percentage of esterified DTX2 than OA in mussels, where the proportion of esterified OA was less than 50%. The same pattern was also observed in Galicia (NW Spain) [153].

Pectenotoxins are also transformed inside bivalves. The most frequent transformation seems to be the opening of the macrolactone ring to produce the seco-acids corresponding to each toxin [46,48,52,59,60,61,130,132,145,152,154,155,156,157,158,159]. It is very likely that this transformation takes place during digestion, as found by MacKenzie et al. [115] in *Perna canaliculus* and other species. Notwithstanding, the transformation from pectenotoxins to their corresponding seco-acids is a species- and toxin- dependent process. The Japanese scallop *Patinopecten yessoensis*, for example, does not hydrolyze the macrolactone ring of PTX2, and consequently it does not generate seco-acids [49,160,161]. There are other bivalve species which have been found to be unable to transform PTX2 into its seco-acid when this toxin was incubated with homogenates of their hepatopancreas or other organs [115]. While the enzyme isolated from *Perna canaliculus* can hydrolyze PTX2 and PTX1, it does not have the same capability for some of their stereoisomers, such as PTX2b and c, and with other compounds of the same group, as PTX6 and PTX11 [45]. PTX12 seco-acids also seem to be less readily formed than those of PTX2 in blue mussels from Norway, but not in the cockle *Cerastoderma edule* from the same location (at least in some cases) [46].

*Patinopecten yessoensis* transforms pectenotoxins in a significantly different way (Figure 7). This species performs a series of successive oxidations of C-43. From PTX2, its hydroxy (PTX1), its aldehyde (PTX3), and finally its carboxylic derivative (PTX6) are formed [49,162]. As far as we know, this sequential oxidation route of PTX2 has only be found in *Patinopecten yessoensis*, and it has the additional peculiarity that it does not take place *in vitro* by incubation with digestive gland extracts [162]. However, the possibility that this process could take place in other bivalve species cannot be ruled out.

The seco-acid of PTX2, at least in some bivalves, undergoes an esterification with fatty acids, as happens with other lipophilic compounds, like okadaic acid and dinophysistoxins 1 and 2 [134] or steroids [163,164]. Three types of esters have been described, depending on the position of the esterified hydroxyl group: C-11, C-37, and C-33 [165]. These esterified forms could be found in some bivalves in a noticeable proportion in relation to PTX2 and PTX2sa [132]. Like the 7-O-acyl esters of the okadaic acid group, several fatty acids may be involved and the mechanism could also be a trans-esterification in which Coenzyme A is involved. In the digestive gland of mussels (*M. galloprovincialis*), an overexpression of genes related to the Coenzyme A activity has been found after exposure to the OA-producing organism *Prorocentrum lima* [166].

## 7. Depuration

Lipophilic toxins do not remain in bivalves indefinitely. They are eliminated from their organs (depurated) at rates that are species- and toxin-dependent. During the intoxication phase, toxins are stored into two main compartments: (a) The outer part of the digestive system (stomach, gut, digestive diverticula), and (b) inside the cells of different organs, mainly the digestive gland. During the depuration phase, shortly after the supply of toxic organisms ceased, the first compartment loses most of its importance, because it includes only the toxins that are being released with feces. Obviously, the mechanisms involved in the elimination of the toxins from each of these two compartments would be completely different. In the case of the first compartment, depuration consists only (or almost only) of the evacuation of the toxins and/or of the particles containing them from the lumen of the digestive organs. In this case, the velocity of the depuration would be related to the rate of renewal of the digestive system, and therefore to the gut passage time, which in turn, is related to the volume of the ingested material. Neither the renewal rate nor the forms in which the toxins are present are expected to be the same in the digestive diverticula and the remaining parts of the digestive system. The digestive diverticula receive material that have already been processed in the stomach and which have been subjected to post-ingestive selection. On the contrary, the stomach and gut contain materials that are unprocessed, are being processed, or have been negatively selected due to their characteristics and/or because of an excess amount of food to be processed. Typically, gut content is renewed within hours, but renewing the diverticula content takes days.

Once the toxins are inside the cells, the depuration mechanisms involved are not very well known. In the okadaic acid group at least, the degradation of the main toxin structural backbone does not seem to be important in light of the existing mass balance studies [64]. As far as we know, no mass balance of the pectenotoxins in the bivalves has been carried out, in part because of the methodological difficulties entailed in quantifying seco-acid esters. Hence, the possibility of the degradation of these toxins cannot be ruled out. In fact, the formation of seco-acids could be considered a degradation of the toxin as the structure is substantially modified by opening the macrolactone cycle and its toxicity is lost.

Therefore, it seems that efflux from the cells would be the main process involved in depuration. Efflux by means of passive diffusion is unlikely because, if that mechanism were important, no accumulation of the toxins would take place. Thus, active efflux through the plasma membrane would take place. This can be done by means of protein membrane transporters or by vesicular transport. In the first case, a number of transporters may be involved, but only a few have been studied. Martínez-Escauriaza [167] and Lozano [168] found in mussels exposed to okadaic acid, an overexpression of genes that codify for membrane transporters, more precisely for a Multidrug Resistance Protein (MDR1, P-glycoprotein) and a Multidrug Resistance-Related Protein (MRP2), both of the ATP-Binding Cassette (ABC) type, which, as commented above, are related to the transport of excess cholesterol and involved in the elimination of multiple xenobiotics from bivalve cells [169,170,171,172,173,174,175,176]. Huang et al. [177] found that the genes that codify for a p-glycoprotein (MDR type) were overexpressed in the mussel *Perna viridis* after its exposure to the OA-producing dinoflagellate *Prorocentrum lima*. Notwithstanding, some specific inhibitors of the activity of the equivalent protein in humans did not increase the amount of OA accumulated by the mussels, which led the authors to suggest that MRP-type proteins could be involved in the efflux of OA. It should be taken into account that inhibitors, known to be effective in human transporter proteins, might be ineffective in their bivalve homologues [170].

The acylation of the molecules of the OA group seems to be an important step in depuration (with the exception of short-term depuration), as most toxins found in bivalve feces are conjugated with fatty acids [64] (+additional unpublished information). This depuration route holds true even for species with a relatively low acylation capability for these toxins, such as the mussel *Mytilus galloprovincialis*, and suggests that the main route for depuration is selective enough to exclude the free forms of the toxins, which suggests that it includes a selective transporter. In fact, from that mussel, DTX2 which esterifies to a lower percentage than OA, depurates more slowly [114,130,145,152].

**Figure 8:**
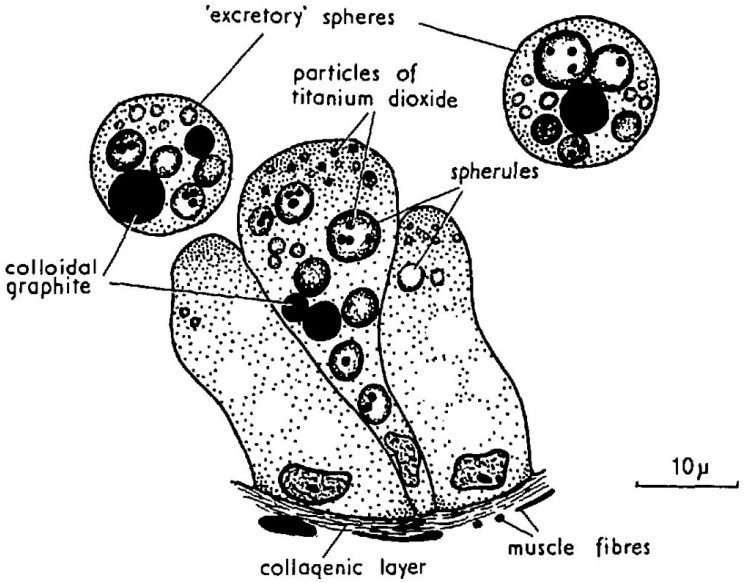
Cells of a digestive tubule after being fed with particles of titanium oxide and colloidal graphite showing the formation and expulsion of excretory spheres containing these materials. Reproduced with permission from Owen [98], published by Company of Biologists 1955.

**Figure 9:**
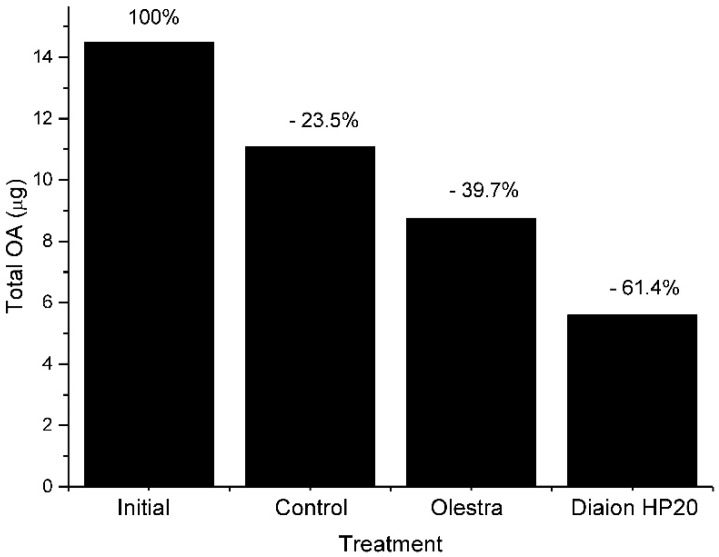
Content in okadaic acid of mussels at the start of the depuration period and after one week. Initial = start of the experiment. Control, Olestra, and Diaion HP20 = after one week being fed with *Tetraselmis suecica* (control), supplemented with Olestra and Diaion HP20.

Vesicular transport could also contribute to depuration. The formation of excretion spheres is a common mechanism of digestive cells to eliminate unassimilated substances (Figure 4 and Figure 8), and we have observed that feeding toxic mussels with substances which bind OA and that cannot be easily digested, like Diaion HP-20 (a synthetic resin) or Olestra (a polyester of sucrose with fatty acids, from Procter and Gamble) substantially accelerated the depuration velocity [64] (Figure 9).

Suárez-Ulloa et al. [166] found that genes related to vesicle-mediated transport are overexpressed in the mussel digestive gland after exposure to the OA producer dinoflagellate *Prorocentrum lima*, which could also support our findings with Diaion and Olestra.

The depuration rates of these toxins are also dependent on the bivalve species and the toxin. It is difficult to extract reliable depuration rates from the literature because they have been obtained in different ways, and in many cases, do not consider all the processes that could affect the amount of a particular toxin in the bivalve body, for instance: (a) In some cases, the change in the toxin burden of the bivalves was used to estimate the rates; however, in many other instances, toxin concentration was used, which means that the estimates are affected by changes in body weight. (b) The whole body was used in some cases, and the digestive gland alone in others. (c) The total amount of a toxin or a particular form of the toxins has also been used. In the former case, the estimated depuration rate is the real depuration rate, but if a particular form of a toxin (free form, for example) is used, then the depuration rate obtained is only apparent because the actual rate is increased by the loss of that form of the toxin—not only by depuration but also by transformation to other forms (for example to acyl-derivatives). Moreover, it is decreased by the transformation of other forms to it (for example from diol-esters to OA) (see some examples in Figure 8, in which some forms of the toxins that do not depurate in the model, have “apparent” depuration rates higher than the forms that are actually depurated). (d) In the cases in which depuration was estimated from bioassay or immunoassay data, the estimates are affected by the toxin profile and its changes. The following data should therefore be considered rough approximations.

In general, the elimination of OA, usually estimated by fitting a first-order exponential decay, is relatively fast in all molluscs. In cultured Galician mussels *Mytilus galloprovincialis*, average depuration rates were around 0.17 to 0.07 day^−1^ or even less at the final part of the depuration phase [44,114,178,179]; 0.19 in *M. galloprovincialis* in the Adriatic sea [180]; and 0.13 in Portugal [130]; approx. 0.13 for Briton and Mediterranean mussels, respectively [181]; 0.05 for *M. edulis* from Norway [145] and 0.13 from Denmark [182]; between 0.07 and 0.17 for *Donax trunculus* [130,183], 0.22 for *Spisula solida* [130], and 0.23 for *Perna viridis* [184].

In general, the estimates of the depuration rates for DTX1 and DTX2 are equal to or lower than for OA [114,145,152]. In *Mytilus galloprovincialis* and *Donax trunculus*—species with low esterification rates—DTX1 and DTX2 are depurated more slowly than OA, which also seems to be true for species with a moderate esterification capability, such as the European oyster *Ostrea edulis* [145]. In several species with high esterification rates, like the cockle *Cardium edule* and others, OA and DTX2 appear to be depurated at similar rates [130,152]. The most likely reason is that these toxins are mostly (after the first steps) depurated as esters, and considering that depuration is proportional to the concentration of the toxin to be depurated, a lower proportion of esters leads to a lower depuration rate.

Very few studies have examined the depuration of esters. Vale [130,152] estimated the depuration rates of OA and DTX2 esters to be higher than those of their free form counterparts, but the opposite was found by Lindegarth et al. [145]. This could be explained because the number of accumulated esters is determined by the balance between esterification and depuration, and consequently, the estimated depuration is only “apparent” and not the real one.

The estimation of the depuration of pectenotoxins is even more inaccurate than that of the toxins of the okadaic acid group because it is impossible to measure the total toxin. In toxins of the OA group, it is possible to transform all chemical forms into free toxins by hydrolysis, but this is not possible with pectenotoxins due to their instability under extreme pH conditions. For example, the estimates of the depuration of PTX2 are overestimated because it is simultaneously depurated and transformed into PTX2sa. The estimates corresponding to PTX2sa are on the one hand, overestimated because it is transformed into PTX2sa-acyl esters, and on the other, underestimated because it derives from PTX2. Even if all these steps are combined in a model, it would be difficult to obtain a correct estimate because it is not possible to quantify all PTX2-acyl esters due to the huge number of possible combinations of fatty acids and locations in the molecule of the esterified hydroxyls.

The “apparent” depuration rate of PTX2 was estimated to be 0.09 day^−1^ for the Norwegian blue mussel *Mytilus edulis* and the flat oyster *Ostrea edulis* [145]. In another mussel, *M. galloprovincialis*, in Portugal, the estimated rate was much higher (0.6–1.1 day^−1^), as was the case of the cockle *Cerastoderma edule* (1–3 day^−1^) [152] and the Chilean surf clam *Mesodesma donacium* [132]. In the Norwegian and Portuguese species, the “apparent” depuration rates of PTX2 were higher than those of OA.

The “apparent” depuration rate of PTX2sa in the flat oyster and *M. edulis* from Norway were similar to that of PTX2 (0.1 and 0.09 day^−1^, respectively) [145]. In the two species studied in Portugal, *C. edule* depurated at a slower rate (0.38 day^−1^) and *M. galloprovincialis* at a similar rate (1.04 day^−1^) [152]. In *Mesodesma donacium* the “apparent” depuration rate showed a decreasing trend with the degree of biotransformation, ranging from 0.3 day^−1^ for PTX2 to 0.2 day^−1^ for palmytoyl-PTX2sa, with an intermediate value of 0.23 day^−1^ for PTX2sa [132].

## 8. Accumulation Kinetics and Modeling

Different models have been used to describe the accumulation kinetics of lipophilic and hydrophilic toxins [185]. For toxin acquisition, the simplest approach assumes a constant feeding rate (K), and a toxin uptake that depends on the feeding rate, the toxin content of the water (TCW), and the absorption efficiency

dTox/dt = K · TCW · (AE)(1)

where TCW can be computed by multiplying the toxic cell concentration in water (Cell_water_) by the toxin content per cell (Tox_cell)_

dTox/dt = K · Cell_water_ · Tox_cell_ · AE (2)

When, after entering the digestive gland, toxins are distributed to other organs or tissues, a multicompartment (usually a two-compartment) model could be used, where the main compartment (compartment 1) acquires the toxin and then it loses a part to the second compartment. In such a case, losses are usually assumed to be proportional to the amount or concentration of toxin

dTox_1_/dt = K · Cell_water_ · Tox_cell_ · AE − TR_1-2_·Tox_1_ (3)

dTox_2_/dt = + TR_1-2_·Tox_1_ (4)

where subindices refer to the compartment and TR is the Transfer Rate between compartments.

When large differences are found in cell concentration in the water, then it might be necessary to express AE (absorption efficiency) as a function of the available cell (or particle) volume which determines the gut passage time (GPT), and consequently the AE, and even to express the feeding rate K as a function of the cell or seston concentration (see Section 2 and Section 3).

When several toxins or toxin derivatives are present, including biotransformations in the kinetic models is mandatory. For example, if diol-esters or sulphated OA or DTXs derivatives (okadaates) are present in *Dinophysis* cells, the free toxins are going to be released and the time course of their abundance cannot be correctly described without transformations. This could explain the anomalies in the accumulation kinetics found by Svensson [186]. Fernández et al. [114] and Moroño et al. [44] included the transformation of these kinds of toxins into free forms, thus improving the model fitting and obtaining what appears to be more realistic estimates of different rates in the model. In OA and Okadaates, the equations would be:

dOA = K · Cell_water_ · OA_cell_ · AE + HR · Okadaates (5)

dOkadaates/dt = K · Cell_water_ · Okadaates_cell_ · AE − HR · Okadaates (6)

where OA_cell_ and Okadaates_cell_ are the concentrations of OA and Okadaates in the cells, and HR is the rate of hydrolysis of Okadaates into OA.

Needless to say, several toxins, derivatives, and compartments could be included.

After the first steps of toxin acquisition, toxin losses due to depuration and/or metabolic transformations of the compounds start to be quantitatively important and should be included in the models. Both biotransformations (formation of 7-O-acyl derivatives (“DTX3”), for example) and depuration are usually assumed to be dependent on the amount (or concentration) of the accumulated toxin. The system of Equations (5) and (6) should be modified to include these two components. Assuming that only 7-O-acyl esters are eliminated, the equation system would be the following:

dOA = K · Cell_water_ · OA_cell_ · AE + HR · Okadaates − AR · OA (7)

dOkadaates/dt = K · Cell_water_ · Okadaates_cell_ · AE − HR · Okadaates (8)

dDTX3 = + AR · OA − DR · DTX3(9)

where DR is the depuration rate of “DTX3”.

**Figure 10:**
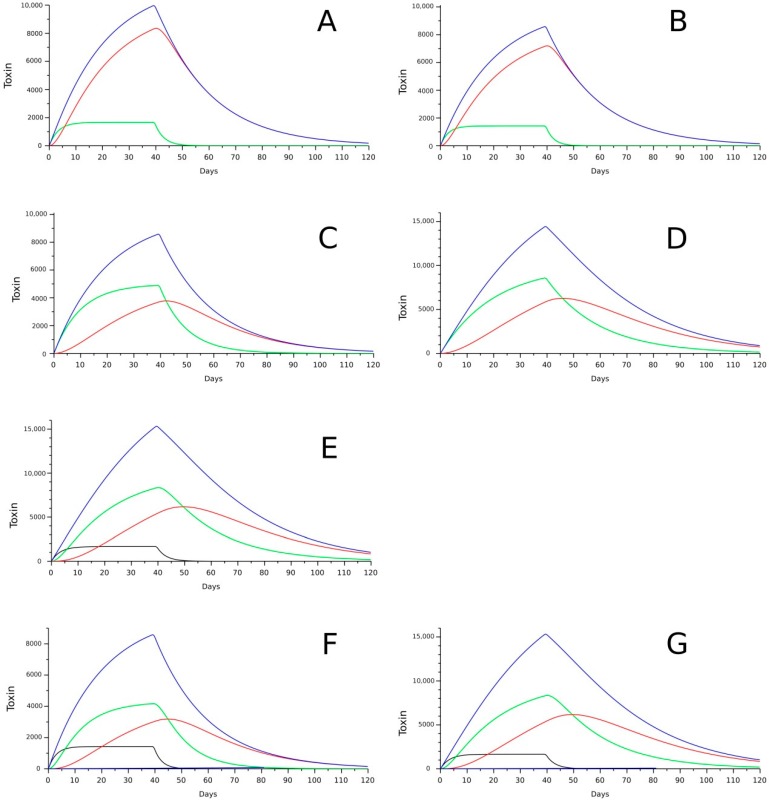
Models of the kinetics of OA and “DTX3” (**A**–**D**), the previous ones plus “DTX5” (**E**), and PTX2, PTX2sa and its esters (**F**,**G**), after 40 days of intoxication (with constant cell abundance in the environment) and 80 days of depuration. The blue line represents total toxin, black is “DTX5” or PTX2, green shows OA or PTX2sa and red, DTX3 or PTX2sa esters. Kinetics with high acylation rate (=0.3 day^−1^) with only DTX3 depuration (**A**), and with OA and DTX3 depuration at the same rate (**B**). Kinetics with low acylation rate (0.05 day-1) with only DTX3 depuration (**C**), and with OA and DTX3 depuration at the same rate as in A and B (**D**). Same as C with input of OA and “DTX5” (50%) (**E**). With depuration of the three forms of PTX2 (**F**) and with depuration of only PTX2sa esters (**G**).

It is clearly necessary to know the toxin forms that are depurated to correctly formulate a model. In the toxins of the OA group, the 7-O-acyl esters appear to be the main toxin form that is depurated, but in the case of pectenotoxins no information is available. Noticeable differences in the kinetics could derive from the routes modeled, as can be observed in some examples in Figure 10.

In the initial steps of depuration, when the undigested toxin stored in the digestive system is quantitatively important, it could be necessary to include an additional compartment and reformulate the models to fit its kinetics. Some possible approaches have been suggested (for particulate matter) by Penry [187].

The build-up of biomass can also be included in the models, thus allowing in this way to describe and predict the allometric changes during the time-course of toxin accumulation.

Recently, a DEB (Dynamic Energy Budgets) model was developed for PSP toxins in the Pacific oyster [188]. Models of this kind include the main metabolic processes of bivalves (including spawning) and would be especially useful when long-term simulations are needed.

## 9. Perspectives

Many areas still need considerable efforts to gather the knowledge that would facilitate the understanding and prediction of the accumulation of toxins produced by *Dinophysis* in bivalve molluscs. When dealing with toxin acquisition, it is necessary to evaluate the effects of *Dinophysis* populations on filtration and on the efficiencies of pre- and post-ingestive selection, as well as the precise mechanism involved in the toxin uptake by the bivalve cells. The mechanisms of depuration for the different toxins, and their interconnection with biotransformation, should also be studied in depth. The use of transcriptomic methodology is promising, but currently, the complexity of the results obtained, together with the lack of knowledge of the precise functions of proteins with or without mammal homologues, makes it difficult to obtain solid and interpretable results. Linking molluscan genes (especially those that codify for membrane transporters) to their actual function would lead to a considerable advance in the elucidation of the depuration mechanisms. It is also important to know which forms of the toxins are eliminated from the bivalves, since they condition not only the possible depuration mechanisms, but also the correct kinetics that should be modeled to obtain a good prediction capability.

In addition to allowing for the development of more precise predictive models, a good knowledge of the mechanisms involved in the accumulation of toxins from *Dinophysis*, would make it easier to develop genetic selection programs, to obtain bivalves with a reduced ability to acquire toxins or with an increased ability to eliminate them. It would also allow development of effective depuration treatments for bivalve species with high commercial value.

## References

[1] Yasumoto T., Oshima Y., Yamaguchi M. (1978). Occurrence of a new type of shellfish poisoning in the Tohoku district. Bull. Jpn. Soc. Sci. Fish..

[2] Yasumoto T., Oshima Y., Yamaguchi M., Taylor D.L., Seliger H.W. (1979). Occurrence of a new type of toxic shellfish in Japan and chemical properties of the toxin. Toxic Dinoflagellate Blooms.

[3] Murata M., Shimatani M., Sugitani H., Oshima Y., Yasumoto T. (1982). Isolation and structural elucidation of the causative toxin of diarrhetic shellfish poisoning. Bull. Jpn. Soc. Sci. Fish..

[4] Tachibana K., Scheuer P., Tsukitani Y., Kikuchi H., Enden V., Clardy J., Gopichand Y., Schmitz F. (1981). Okadaic acid, a cytotoxic polyether from two marine sponges of the genus *Halichondria*. J. Am. Chem. Soc..

[5] Tangen K. (1983). Shellfish poisoning and the ocurrence of potentially toxic dinoflagellates in Norwegian waters. Sarsia.

[6] Kat M. (1983). Diarrhetic mussel poisoning in the Netherlands related to the dinoflagellate *Dinophysis acuminata*. Antonie Van Leeuenhoek.

[7] Kumagai M., Yanagi T., Murata M., Yasumoto T., Kat M., Lassus P., Rodriguez-Vázquez J.A. (1986). Okadaic acid as the causative toxin of Diarrhetic Shellfish Poisoning in Europe. Agric. Biol. Chem..

[8] Van Egmond H.P., Aune T., Lassus P., Speijers G.J.A., Waldock M. (1993). Paralytic and diarrhoeic shellfish poisons: Occurrence in Europe, toxicity, analysis and regulation. J. Nat. Toxins.

[9] James K.J., Carey B., O′Halloran J., van Pelt F.N.A.M., Skrabakova Z. (2010). Shellfish toxicity: Human health implications of marine algal toxins. Epidemiol. Infect..

[10] EFSA Panel on Contaminants in the Food Chain (2008). Opinion of the Scientific Panel on Contaminants in the Food chain on a request from the European Commission on marine biotoxins in shellfish okadaic acid and analogues. EFSA J..

[11] EFSA Panel on Contaminants in the Food Chain (2009). Scientific Opinion of the Panel on Contaminants in the Food Chain on a request from the European Commission on Marine Biotoxins in Shellfish—Summary on regulated marine biotoxins. EFSA J..

[12] Commission Regulation (EU) No 15/2011. Amending Regulation (EC) No 2074/2005 as Regards Recognised Testing Methods for Detecting Marine Biotoxins in Live Bivalve Molluscs. https://eur-lex.europa.eu/LexUriServ/LexUriServ.do?uri=OJ:L:2011:006:0003:0006:EN:PDF.

[13] EFSA Panel on Contaminants in the Food Chain (2009). Marine biotoxins in shellfish—Pectenotoxin group: Marine biotoxins in shellfish—Pectenotoxin group. EFSA J..

[14] Fernández M.L., Shumway S.E., Blanco J., Hallegraeff G.M., Anderson A.D., Anderson D.M. (2003). Management of shellfish resources. Manual on Harmful Marine Microalgae.

[15] Blanco J., Correa J., Muñíz S., Mariño C., Martín H., Arévalo F. (2013). Evaluación del impacto de los métodos y niveles utilizados para el control de toxinas en el mejillón. Revista Galega dos Recursos Mariños.

[16] Lee J.S., Igarashi T., Fraga S., Dahl E., Hovgaard P., Yasumoto T. (1989). Determination of diarrhetic shellfish toxins in various dinoflagellate species. J. Appl. Phycol..

[17] Fux E., Smith J.L., Tong M., Guzmán L., Anderson D.M. (2011). Toxin profiles of five geographical isolates of *Dinophysis* spp. from North and South America. Toxicon.

[18] Uribe J.C., García C., Rivas M., Lagos N. (2001). First report of diarrhetic shellfish toxins in Magellanic fjord, Southern Chile. J. Shellfish Res..

[19] Taylor M., McIntyre L., Ritson M., Stone J., Bronson R., Bitzikos O., Rourke W., Galanis E., Team O. (2013). Outbreak of Diarrhetic Shellfish Poisoning Associated with Mussels, British Columbia, Canada. Mar. Drugs.

[20] García-Mendoza E., Sánchez-Bravo Y.A., Turner A., Blanco J., O’Neil A., Mancera-Flores J., Pérez-Brunius P., Rivas D., Almazán-Becerril A., Peña-Manjarrez J.L. (2014). Lipophilic toxins in Mediterranean Mussels from the northwest coast of Baja California, México. Toxicon.

[21] Carmody E.P., James K.J., Kelly S.S. (1996). Dinophysistoxin-2: The predominant diarrhetic shellfish toxin in Ireland. Toxicon.

[22] Blanco J., Fernández M.L., Mariño J., Reguera B., Miguez A., Maneiro J., Cacho E., Martínez A., Lassus P., Arzul G., Erard-Le Denn E., Gentien P., Marcaillou-Le Baut C. (1995). From *Dinophysis* spp. toxicity to DSP outbreaks: Apreliminary model of toxin accumulation in mussels. Harmful Marine Algal Blooms.

[23] Gago A., Rodriguez-Vázquez J.A., Thibault P., Quilliam M.A. (1996). Simultaneus occurrence of diarrhetic and paralytic shellfish poisoning toxins in Spanish mussels in 1993. Nat. Toxins.

[24] Vale P., Sampayo M.A., Yasumoto T., Oshima Y., Fukuyo Y. (1996). DTX-2 in Portuguese bivalves. Harmful and Toxic Algal Blooms.

[25] Aune T., Larsen S., Aasen J.A.B., Rehmann N., Satake M., Hess P. (2007). Relative Toxicity of Dinophysistoxin-2 (DTX-2) Compared With Okadaic Acid, Based on Acute Intraperitoneal Toxicity in Mice. Toxicon.

[26] Dhanji-Rapkova M., O′Neill A., Maskrey B.H., Coates L., Teixeira Alves M., Kelly R.J., Hatfield R.G., Rowland-Pilgrim S.J., Lewis A.M., Algoet M. (2018). Variability and profiles of lipophilic toxins in bivalves from Great Britain during five and a half years of monitoring: Okadaic acid, dinophysis toxins and pectenotoxins. Harmful Algae.

[27] Elgarch A., Vale P., Rifai S., Fassouane A. (2008). Detection of Diarrheic Shellfish Poisoning and Azaspiracid Toxins in Moroccan Mussels: Comparison of the LC-MS Method with the Commercial Immunoassay Kit. Mar. Drugs.

[28] Armi Z., Turki S., Trabelsi E., Ceredi A., Riccardi E., Milandri A. (2012). Occurrence of diarrhetic shellfish poisoning (DSP) toxins in clams (*Ruditapes decussatus*) from Tunis north lagoon. Environ. Monit. Assess..

[29] Pavela-Vrancic M., Mestrovic V., Marasovic I., Gillman M., Furey A., James K.J. (2002). DSP toxin profile in the coastal waters of the central Adriatic Sea. Toxicon.

[30] Vale P., Sampayo M.A. (2000). Dinophysistoxin-2: A rare diarrhoeic toxin associated with *Dinophysis acuta*. Toxicon.

[31] Draisci R., Giannetti L., Lucentini L., Marchiafava C., James K.J., Bishop A.G., Healy B.M., Kelly S.S. (1998). Isolation of a new okadaic acid analog from phytoplankton implicated in diarrhetic shellfish poisoning. J. Chromatogr..

[32] Hu T., Doyle J., Jackson D.M., Marr J., Nixon E., Pleasance S., Quilliam M.A., Walter J.A., Wright J.L.C. (1992). Isolation of a new diarrhetic shellfish poison from Irish mussels. J. Chem. Soc. Chem. Commun..

[33] Hu T., Marr J., de Freitas A.S.W., Quilliam M.A., Walter J.A., Wright J.L.C., Pleasance S. (1992). New diol esters isolated from cultures of *Prorocentrum lima* and *Prorocentrum concavum*. J. Nat. Prod..

[34] Hu T., Curtis J.M., Walter J.A., McLachlan J.L., Wright J.L.C. (1995). Two new water-soluble dsp toxin derivatives from the dinoflagellate *Prorocentrum maculosum*: Possible storage and excretion products. Tetrahedron Lett..

[35] Hu T., Curtis J.M., Walter J.A., Wright J.L.C. (1995). Identification of DTX-4, A New water-soluble phosphatase inhibitor from the toxic dinoflagellate *Prorocentrum lima*. J. Chem. Soc. Chem. Commun..

[36] Hu T.M., LeBlanc P., Burton I.W., Walter J.A., McCarron P., Melanson J.E., Strangman W.K., Wright J.L.C. (2017). Sulfated diesters of okadaic acid and DTX-1: Self-protective precursors of diarrhetic shellfish poisoning (DSP) toxins. Harmful Algae.

[37] Pan L., Chen J.H., Shen H.H., He X.P., Li G.J., Song X.C., Zhou D.S., Sun C.J. (2017). Profiling of Extracellular Toxins Associated with Diarrhetic Shellfish Poison in *Prorocentrum lima* Culture Medium by High-Performance Liquid Chromatography Coupled with Mass Spectrometry. Toxins.

[38] Torgersen T., Miles C.O., Rundberget T., Wilkins A.L. (2008). New Esters of Okadaic Acid in Seawater and Blue Mussels (*Mytilus edulis*). J. Agric. Food Chem..

[39] Cruz P.G., Daranas A.H., Fernandez J.J., Souto M.L., Norte M. (2006). DTX5c, a new OA sulphate ester derivative from cultures of *Prorocentrum belizeanum*. Toxicon.

[40] Suzuki H., Beuzenberg V., MacKenzie A.L., Quilliam M.A. (2004). Discovery of okadaic acid esters in the toxic dinoflagellate *Dinophysis acuta* from New Zealand using liquid chromatography/tandem mass spectrometry. Rapid Commun. Mass Spectrom..

[41] Miles C.O., Wilkins A.L., Hawkes A.D., Jensen D.J., Cooney J.M., Larsen K., Petersen D., Rise F., Beuzenberg V., MacKenzie A.L. (2006). Isolation and identification of a cis-C-8-diol-ester of okadaic acid from Dinophysis acuta in New Zealand. Toxicon.

[42] Pizarro G., Paz B., Franco J., Suzuki T., Reguera B. (2008). First detection of Pectenotoxin-11 and confirmation of OA-D8 diol-ester in *Dinophysis acuta* from European waters by LC-MS/MS. Toxicon.

[43] Campbell L., Olson R., Sosik H.M., Abraham A., Henrichs D.W., Hyatt C., Buskey E.J. (2010). First harmful *Dinophysis* (Dinophyceae, Dinophysiales) bloom in the U.S. is revealed by automated imaging flow cytometry. J. Phycol..

[44] Moroño A., Arevalo F., Fernandez M., Maneiro J., Pazos Y., Salgado C., Blanco J. (2003). Accumulation and transformation of DSP toxins in mussels *Mytilus galloprovincialis* during a toxic episode caused by *Dinophysis acuminata*. Aquat. Toxicol..

[45] Suzuki T., Walter J.A., LeBlanc P., MacKinnon S., Miles C.O., Wilkins A.L., Munday R., Beuzenberg V., MacKenzie L., Jensen D.J. (2006). Identification of Pectenotoxin-11 as 34S-Hydroxypectenotoxin-2, a New Pectenotoxin Analogue in the Toxic Dinoflagellate *Dinophysis acuta* from New Zealand. Chem. Res. Toxicol..

[46] Miles C.O., Wilkins A.L., Samdal I.A., Sandvik M., Petersen D., Quilliam M.A., Naustvoll L.J., Jensen D.J., Cooney J.M. (2004). A novel pectenotoxin, PTX-12, in *Dinophysis* spp. and shellfish from Norway. Chem. Res. Toxicol..

[47] Draisci R., Lucentini L., Giannetti L., Boria P., Poletti R. (1996). First report of pectenotoxin-2 (PTX-2) in algae (*Dinophysis fortii*) related to seafood poisoning in Europe. Toxicon.

[48] Daiguji M., Satake M., James K.J., Bishop A., MacKenzie L., Naoki H., Yasumoto T. (1998). Structures of new pectenotoxin analogs, pectenotoxin-2 seco acid and 7-epi-pectenotoxin-2 seco acid, isolated from a dinoflagellate and greenshell mussels. Chem. Lett..

[49] Suzuki T., Mitsuya T., Matsubara H., Yamasaki M. (1998). Determination of pectenotoxin-2 after solid-phase extraction from seawater and from the dinoflagellate *Dinophysis fortii* by liquid chromatography with electrospray mass spectrometry and ultraviolet detection. Evidence of oxidation of pectenotoxin-2 to pectenotoxin-6 in scallop. J Chomatogr. A.

[50] Suzuki T., Miyazono A., Baba K., Sugawara R., Kamiyama T. (2009). LC-MS/MS analysis of okadaic acid analogues and other lipophilic toxins in single-cell isolates of several *Dinophysis* species collected in Hokkaido, Japan. Harmful Algae.

[51] Suzuki T., Miyazono A., Okumura Y., Kamiyama T. LC-MS/MS Analysis of Lipophilic Toxins in Japanese *Dinophysis* Species. http://www.pices.int/publications/presentations/PICES_15/Ann15_W4/W4_Suzuki.pdf.

[52] James K.J., Bishop A.G., Draisci R., Palleschi L., Marchiafava C., Ferretti E., Satake M., Yasumoto T. (1999). Liquid chromatographic methods for the isolation and identification of new pectenotoxin-2 analogues from marine phytoplankton and shellfish. J. Chromatogr..

[53] Sasaki K., Takizawa A., Tubaro A., Sidari L., Loggia R.D., Yasumoto T. (1999). Fluorometric analysis of pectenotoxin-2 in microalgal samples by high performance liquid chromatography. Nat. Toxins.

[54] Fernández M.L., Reguera B., González-Gil S., Míguez A. (2006). Pectenotoxin-2 in single-cell isolates of *Dinophysis caudata* and *Dinophysis acuta* from the Galician Rías (NW Spain). Toxicon.

[55] Kamiyama T., Suzuki T. (2009). Production of dinophysistoxin-1 and pectenotoxin-2 by a culture of *Dinophysis acuminata* (Dinophyceae). Harmful Algae.

[56] Nagai S., Suzuki T., Nishikawa T., Kamiyama T. (2011). Differences in the production and excretion kinetics of okadaic acid, dinophysistoxin-1, and pectenotoxin-2 between cultures of *Dinophysis acuminata* and *Dinophysis fortii* isolated from western Japan. J. Phycol..

[57] MacKenzie L., Beuzenberg V., Holland P., McNabb P., Suzuki T., Selwood A. (2005). Pectenotoxin and okadaic acid-based toxin profiles in *Dinophysis acuta* and *Dinophysis acuminata* from New Zealand. Harmful Algae.

[58] Fabro E., Almandoz G.O., Ferrari M.E., Hoffmeyer M.S., Pettigrosso R.E., Uibrig R., Krock B. (2015). Co-occurrence of Dinophysis tripos and pectenotoxins in Argentinean shelf waters. Harmful Algae.

[59] Vale P., Sampayo M.A. (2002). Pectenotoxin-2 seco acid, 7-epi-pectenotoxin-2 seco acid and pectenotoxin-2 in shellfish and plankton from Portugal. Toxicon.

[60] Fernández-Puente P., Fidalgo Sáez M.J., Hamilton B., Furey A., James K.J. (2004). Studies of polyether toxins in the marine phytoplankton, *Dinophysis acuta*, in Ireland using multiple tandem mass spectrometry. Toxicon.

[61] Blanco J., Álvarez G., Uribe E. (2007). Identification of pectenotoxins in plankton, filter feeders, and isolated cells of a *Dinophysis acuminata* with an atypical toxin profile from Chile. Toxicon.

[62] Nielsen L.T., Krock B., Hansen P.J. (2012). Effects of light and food availability on toxin production, growth and photosynthesis in *Dinophysis acuminata*. Mar. Ecol. Prog. Ser..

[63] Li A., Li M., Qiu J., Song J., Ji Y., Hu Y., Wang S., Che Y. (2018). Effect of Suspended Particulate Matter on the Accumulation of Dissolved Diarrhetic Shellfish Toxins by Mussels (*Mytilus galloprovincialis*) under Laboratory Conditions. Toxins.

[64] Rossignoli A.E. (2011). Acumulación de Toxinas DSP en el mejillón *Mytilus galloprovincialis*. Ph.D. Thesis.

[65] Nam K.Y., Hiro M., Kimura S., Fujiki H., Imanishi Y. (1990). Permeability of a non-TPA-type tumor promoter, okadaic acid, through lipid bilayer membrane. Carcinogenesis.

[66] Daranas A.H., Cruz P.G., Creus A.H., Norte M., Fernández J.J. (2007). Self-assembly of okadaic acid as a pathway to the cell. Org. Lett..

[67] Jauffrais T., Kilcoyne J., Herrenknecht C., Truquet P., Sechet V., Miles C.O., Hess P. (2013). Dissolved azaspiracids are absorbed and metabolized by blue mussels (*Mytilus edulis*). Toxicon.

[68] Dame R.F. (2013). Bivalve Filter Feeders: In Estuarine and Coastal Ecosystem Processes.

[69] Gosling E. (2015). Marine Bivalve Molluscs.

[70] Møhlenberg F., Riisgård H.U. (1978). Efficiency of particle retention in 13 species of suspension feeding bivalves. Ophelia.

[71] Riisgård H.U. (1988). Efficiency of particle retention and filtration rate in 6 species of Northeast American bivalves. Mar. Ecol. Prog. Ser..

[72] Vahl O. (1972). Particle retention and relation between water transport and oxygen uptake in *Chlamys opercularis* (L.) (Bivalvia). Ophelia.

[73] Sobral P., Widdows J. (2000). Effects of increasing current velocity, turbidity and particle-size selection on the feeding activity and scope for growth of Ruditapes decussatus from Ria Formosa, southern Portugal. J. Exp. Mar.Biol. Ecol..

[74] Reguera B., Riobó P., Rodríguez F., Díaz P., Pizarro G., Paz B., Franco J., Blanco J. (2014). Dinophysis Toxins: Causative Organisms, Distribution and Fate in Shellfish. Mar. Drugs.

[75] Iglesias J.I.P., Urrutia M.B., Navarro E., Alvarez-Jorna P., Larretxea X., Bougrier S., Heral M. (1996). Variability of feeding processes in the cockle *Cerastoderma edule* (L.) in response to changes in seston concentration and composition. J. Exp. Mar.Biol. Ecol..

[76] Pitcher G.C., Krock B., Cembella A.D. (2011). Accumulation of diarrhetic shellfish poisoning toxins in the oyster *Crassostrea gigas* and the mussel *Choromytilus meridionalis* in the southern Benguela ecosystem. Afr. J. Mar. Sci..

[77] García-Altares M., Casanova A., Fernández-Tejedor M., Diogène J., De La Iglesia P. (2016). Bloom of *Dinophysis* spp. dominated by *D. sacculus* and its related diarrhetic shellfish poisoning (DSP) outbreak in Alfacs Bay (Catalonia, NW Mediterranean Sea): Identification of DSP toxins in phytoplankton, shellfish and passive samplers. Reg. Stud. Mar. Sci..

[78] Kim J.H., Lee K.J., Suzuki T., Kang Y.S., Ho Kim P., Song K.C., Lee T.S. (2010). Seasonal Variability of Lipophilic Shellfish Toxins in Bivalves and Waters, and Abundance of *Dinophysis* spp. in Jinhae Bay, Korea. J. Shellfish Res..

[79] Kacem I., Bouaïcha N., Hajjem B. (2010). Comparison of okadaic acid profiles in mussels and oysters collected in Mediterranean lagoon, Tunisia. Int.J. Biol..

[80] Comeau L.A., Pernet F., Tremblay R., Bates S.S., LeBlanc A. (2008). Comparison of eastern oyster (*Crassostrea virginica*) and blue mussel (*Mytilus edulis*) filtration rates at low temperatures. Can. Tech. Rep. Fish. Aquat. Sci..

[81] McFarland K., Donaghy L., Volety A.K. (2013). Effect of acute salinity changes on hemolymph osmolality and clearance rate of the non-native mussel, *Perna viridis*, and the native oyster, Crassostrea virginica, in Southwest Florida. Aquat. Invasions.

[82] Cranford P.J., Ward J.E., Shumway S.E., Shumway S.E. (2011). Bivalve filter feeding: Variability and limits of the aquaculture biofilter. Shellfish Aquaculture and the Environment.

[83] Bricelj V.M., Lee J.H., Cembella A.D. (1991). Influence of dinoflagellate cell toxicity on uptake and loss of paralytic shellfish toxins in the northern quahog *Mercenaria mercenaria*. Mar. Ecol. Prog. Ser..

[84] Mafra L., Bricelj V., Ouellette C., Léger C., Bates S. (2009). Mechanisms contributing to low domoic acid uptake by oysters feeding on *Pseudo-nitzschia* cells. I. Filtration and pseudofeces production. Aquat. Biol..

[85] Basti L., Uchida H., Kanamori M., Matsushima R., Suzuki T., Nagai S. (2014). Mortality and pathology of Japanese scallop, *Patinopecten* (*Mizuhopecten*) *yessoensis*, and noble scallop, *Mimachlamys nobilis*, fed monoclonal culture of PTX-producer, *Dinophysis caudata*. Marine and Freshwater Harmful Algae, Proceedings of the 16th International Conference on Harmful Algae, Wellington, New Zealand, Wellington, New Zealand, 27–31 October 2014.

[86] Pillet S., Houvenaghel G., Lassus P., Arzul G., Erard E., Gentien P., Marcaillou C. (1995). Influence of experimental toxification by DSP producing microalgae, *Prorocentrum lima*, on clearance rate in blue mussels *Mytilus edulis*. Harmful Marine Algal Blooms.

[87] Sampayo M.A., Alvito P., Franca S., Sousa I., Granéli E., Sundström B., Edler L., Anderson D.M. (1990). *Dinophysis* spp. toxicity and relation to accompanying species. Toxic Marine Phytoplankton.

[88] Haamer J. (1995). Presence of the phycotoxin okadaic acid in mussel (*Mytilus edulis*) in relation to nutrient composition in a Swedish coastal water. J. Shellfish Res..

[89] Jorgensen C.B. (1990). Bivalve Filter Feeding: Hydrodynamics, Bioenergetics, Physiology and Ecology.

[90] Iglesias J.I.P., Navarro E., Alvarez Jorna P., Armentia I. (1992). Feeding, particle selection and absorption in cockles *Cerastoderma edule* (L.) exposed to variable conditions of food concentration and quality. J. Exp. Mar. Biol. Ecol..

[91] Shumway S.E., Cucci T.L., Lesser M.P., Bourne N., Bunting B. (1997). Particle clearance and selection in three species of juvenile scallops. Aquac. Int..

[92] Shumway S.E., Cucci T.L., Newell R.C., Yentsch C.M. (1985). Particle selection, ingestion, and absorption in filter-feeding bivalves. J. Exp. Mar. Biol. Ecol..

[93] Ward J.E., Shumway S.E. (2004). Separating the grain from the chaff: Particle selection in suspension- and deposit-feeding bivalves. J. Exp. Mar.Biol. Ecol..

[94] Bricelj V.M., Ward J.E., Cembella A.D., MacDonald B.A., Reguera B., Blanco J., Fernández M.L., Wyatt T. (1998). Application of video-endoscopy to the study of bivalve feeding on toxic dinoflagellates. Harmful Algae.

[95] Defossez J.M., Hawkins A.J.S. (1997). Selective feeding in shellfish: Size-dependent rejection of large particles within pseudofaeces from *Mytilus edulis*, *Ruditapes philippinarum* and *Tapes decussatus*. Mar. Biol..

[96] Sidari L., Nichetto P., Cok S., Sosa S., Tubaro A., Honsell G., DellaLoggia R. (1998). Phytoplankton selection by mussels, and diarrhetic shellfish poisoning. Mar. Biol..

[97] Pino-Querido A., Alvarez-Castro J.M., Guerra-Varela J., Toro M.A., Vera M., Pardo B.G., Fuentes J., Blanco J., Martinez P. (2015). Heritability estimation for okadaic acid algal toxin accumulation, mantle color and growth traits in Mediterranean mussel (*Mytilus galloprovincialis*). Aquaculture.

[98] Owen G. (1955). Observations on the Stomach and Digestive Diverticula of the Lamellibranchia: I. The Anisomyaria and Eulamellibranchia. Q. J. Microsc. Sci..

[99] Purchon R.D. (1987). The stomach in the bivalvia. Philos. Trans. R. Soc. Lond..

[100] Hawkins A.J.S., Navarro E., Iglesias J.I.P. (1990). Comparative allometries of gut-passage time, gut content and metabolic faecal loss in *Mytilus edulis* and *Cerastoderma edule*. Mar. Biol..

[101] Navarro E., Iglesias J.I.P., Dame R.F. (1993). Infaunal Filter-Feeding Bivalves and the Physiological Response to Short-Term Fluctuations in Food Availability and Composition. Bivalve Filter Feeders.

[102] Moroño A., Franco J., Miranda M., Reyero M.I., Blanco J. (2001). The effect of mussel size, temperature, seston volume, food quality and volume-specific toxin concentration on the uptake rate of PSP toxins by mussels (*Mytilus galloprovincialis* LmK). J. Exp. Mar. Biol. Ecol..

[103] Guéguen M., Lassus P., Laabir M., Bardouil M., Baron R., Séchet V., Truquet P., Amzil Z., Barillé L. (2008). Gut passage times in two bivalve molluscs fed toxic microalgae: *Alexandrium minutum, A. catenella* and *Pseudo-nitzschia calliantha*. Aquat. Living Res..

[104] Brillant M.G., MacDonald B.A. (2000). Postingestive selection in the sea scallop, *Placopecten magellanicus* (Gmelin): the role of particle size and density. J. Exp. Mar. Biol. Ecol..

[105] Bricelj V.M., Bass A.E., Lopez G.R. (1984). Absorption and gut passage time of microalgae in a suspension feeder: an evaluation of the 51Cr: 14C twin tracer technique. Mar. Ecol. Prog. Ser..

[106] Bayne B.L. (1993). Feeding physiology of bivalves: Time-dependence and compensation for changes in food availability. Bivalve Filter Feeders.

[107] Bauder A.G., Cembella A.D. (2000). Viability of the toxic dinoflagellate *Prorocentrum lima* following ingestion and gut passage in the bay scallop *Argopecten irradians*. J. Shellfish Res..

[108] Bauder A.G., Cembella A.D., Bricelj V.M., Quilliam M.A. (2001). Uptake and fate of diarrhetic shellfish poisoning toxins from the dinoflagellate *Prorocentrum lima* in the bay scallop *Argopecten irradians*. Mar. Ecol. Prog. Ser..

[109] Rossignoli A.E., Fernández D., Acosta C.P., Blanco J. (2011). Microencapsulation of okadaic acid as a tool for studying the accumulation of DSP toxins in mussels. Mar. Environ. Res..

[110] Windust A.J., Hu T.M., Wright J.L.C., Quilliam M.A., McLachlan J.L. (2000). Oxidative metabolism by *Thalassiosira weissflogii* (Bacillariophyceae) of a diol-ester of okadaic acid, the diarrhetic shellfish poisoning. J. Phycol..

[111] Quilliam M.A., Hardstaff W.R., Ishida N., McLachlan J.L., Reeves A.R., Ross N.W., Windust A.J., Yasumoto T., Oshima Y., Fukuyo Y. (1996). Production of diarrhetic shellfish poisoning (DSP) toxins by *Prorocentrum lima* in culture and development of analytical methods. Harmful and Toxic Algal Blooms.

[112] Quilliam M.A., Ishida N., McLachlan J.L., Ross N.W., Windust A.J. Analytical Methods for Diarrhetic Shellfish Poisoning (DSP) Toxins and A Study of Toxin Production by Prorocentrum lima in Culture. https://repository.library.noaa.gov/view/noaa/12555/noaa_12555_DS1.pdf?#page=101.

[113] Morton B., Saleuddin A.S.M., Wilbur K.M. (1983). Feeding and digestion in Bivalvia. The Mollusca. Physiology Part 2.

[114] Fernández M.L., Miguez A., Moroño A., Cacho E., Martínez A., Blanco J., Reguera B., Blanco J., Fernández M.L., Wyatt T. (1998). Detoxification of low polarity toxins (DTX3) from mussels *Mytilus galloprovincialis* in Spain. Harmful Algae.

[115] MacKenzie L.A., Selwood A.I., Marshall C. (2012). Isolation and characterization of an enzyme from the Greenshell (TM) mussel *Perna canaliculus* that hydrolyses pectenotoxins and esters of okadaic acid. Toxicon.

[116] Fux E. (2008). Development and Evaluation of Passive Sampling and LC-MS Based Techniques for the Detection and Monitoring of Lipophilic Marine Toxins in Mesocosm and Field Studies. Ph.D. Thesis.

[117] Rossignoli A.E., Blanco J. (2010). Subcellular distribution of okadaic acid in the digestive gland of *Mytilus galloprovincialis*: First evidences of lipoprotein binding to okadaic acid. Toxicon.

[118] Guéguen M., Duinker A., Marcaillou C. A first approach to localizing biotoxins in mussel digestive glands. Proceedings of the 7th International Conference on Molluscan Shellfish Safety.

[119] Moore M.N., Willows R.I. (1998). A model for cellular uptake and intracellular behaviour of particulate-bound micropollutants. Mar. Environ. Res..

[120] Smedes F. (1994). Sampling and partitioning of neutral organic contaminants in surface waters with regard to legislation, environmental quality and flux estimations. Int. J. Environ. Anal. Chem..

[121] Rossignoli A., Blanco J. (2008). Cellular distribution of okadaic acid in the digestive gland of *Mytilus galloprovincialis* (Lamarck, 1819). Toxicon.

[122] Weinstein J.E. (1995). Fine structure of the digestive tubule of the eastern oyster, *Crassostrea virginica* (Gmelin, 1791). J. Shellfish Res..

[123] Jonas A., Vance D.E., Vance J.E. (2002). Lipoprotein structure. Biochemistry of Lipids, Lipoproteins and Membranes.

[124] Blanco J., Mariño C., Martín H., Acosta C.P. (2007). Anatomical distribution of Diarrhetic Shellfish Poisoning (DSP) toxins in the mussel *Mytilus galloprovincialis*. Toxicon.

[125] Marcaillou C., Haure J., Mondeguer F., Courcoux A., Dupuy B., Penisson C. (2010). Effect of food supply on the detoxification in the blue mussel, *Mytilus edulis*, contaminated by diarrhetic shellfish toxins. Aquat. Living Resour..

[126] Edebo L., Lange S., Li X.P., Allenmark S., Lindgren K., Thompson R. (1988). Seasonal, geographic and individual variation of okadaic acid content in cultivated mussels in Sweden. Apmis.

[127] McCarron P., Kilcoyne J., Hess P. (2008). Effects of cooking and heat treatment on concentration and tissue distribution of okadaic acid and dinophysistoxin-2 in mussels (*Mytilus edulis*). Toxicon.

[128] Madigan T.L., Lee K.G., Padula D.J., McNabb P., Pointon A.M. (2006). Diarrhetic shellfish poisoning (DSP) toxins in South Australian shellfish. Harmful Algae.

[129] Hess P., McMahon T., Slattery D., Swords D., Dowling G., McCarron M., Clarke D., Gibbons W., Silke W., O’Cinneide M., Villalba A., Reguera B., Romalde J.L., Beiras R. (2003). Use of LC-MS testing to identify lipophilic toxins, to stablish local trends and interspecies differences and to test the comparability of LC-MS testing with mouse bioassay: An example from the Irish Biotoxin Monitoring Programme 2001. Molluscan Shellfish Safety, Proceedings of the 4th International Conference on Molluscan Shellfish Safety, Xunta De Galicia, Spain, 4–8 June 2002.

[130] Vale P. (2006). Differential Dynamics of Dinophysistoxins and Pectenotoxins, Part II: Offshore Bivalve Species. Toxicon.

[131] Kameneva P.A., Krasheninina E.A., Slobodskova V.V., Kukla S.P., Orlova T.Y. (2017). Accumulation and Tissue Distribution of Dinophysitoxin-1 and Dinophysitoxin-3 in the Mussel *Crenomytilus grayanus* Feeding on the Benthic Dinoflagellate *Prorocentrum foraminosum*. Mar. Drugs.

[132] Blanco J., Álvarez G., Rengel J., Díaz R., Mariño C., Martín H., Uribe E. (2018). Accumulation and Biotransformation of *Dinophysis* Toxins by the Surf Clam *Mesodesma donacium*. Toxins.

[133] Suzuki T., Ota H., Yamasaki M. (1999). Direct evidence of transformation of dinophysistoxin-1 to 7-O-acyl-dinophysistoxin-1 (Dinophysis-3) in the scallop *Patinopecten yessoensis*. Toxicon.

[134] Rossignoli A.E., Fernández D., Regueiro J., Mariño C., Blanco J. (2011). Esterification of okadaic acid in the mussel *Mytilus galloprovincialis*. Toxicon.

[135] Konoki K., Onoda T., Watanabe R., Cho Y., Kaga S., Suzuki T., Yotsu-Yamashita M. (2013). In Vitro Acylation of Okadaic Acid in the Presence of Various Bivalves′ Extracts. Mar. Drugs.

[136] Vale P. (2006). Detailed profiles of 7-O-acyl esters in plankton and shellfish from the Portuguese coast. J. Chomatogr. A.

[137] Quilliam M.A., Vale P., Sampayo M.A.M., Villalba A., Reguera B., Romalde J., Beiras R. (2003). Direct detection of acyl esters of okadaic acid and dinophysistoxin-2 in Portuguese shellfish by LC-MS. Molluscan Shellfish Safety.

[138] Iglesia P., Fonollosa E., Diogène J. (2014). Assessment of acylation routes and structural characterisation by liquid chromatography/tandem mass spectrometry of semi-synthetic acyl ester analogues of lipophilic marine toxins. Rapid Commun. Mass Spectrom..

[139] Suzuki T., Kamiyama T., Okumura Y., Ishihara K., Matsushima R., Kaneniwa M. (2009). Liquid-chromatographic hybrid triple–quadrupole linear-ion-trap MS/MS analysis of fatty-acid esters of dinophysistoxin-1 in bivalves and toxic dinoflagellates in Japan. Fish. Sci..

[140] Torgersen T., Wilkins A.L., Rundberget T., Miles C.O. (2008). Characterization of fatty acid esters of okadaic acid and related toxins in blue mussels (*Mytilus edulis*) from Norway. Rapid Commun. Mass Spectrom..

[141] Marr J., Hu T., Pleasance S., Quilliam M.A., Wright J.L.C. (1992). Detection of new 7-*O*-acyl derivatives of diarrhetic shellfish poisoning toxins by liquid chromatography- mass spectometry. Toxicon.

[142] Vale P. (2010). Profiles of fatty acids and 7-O-acyl okadaic acid esters in bivalves: Can bacteria be involved in acyl esterification of okadaic acid?. Comp. Biochem. Physiol. C Toxicol. Pharmacol..

[143] Trainer V.L., Moore L., Bill B., Adams N., Harrington N., Borchert J., da Silva D., Eberhart B. (2013). Diarrhetic Shellfish Toxins and Other Lipophilic Toxins of Human Health Concern in Washington State. Mar. Drugs.

[144] Torgersen T., Sandvik M., Lundve B., Lindegarth S. (2008). Profiles and levels of fatty acid esters of okadaic acid group toxins and pectenotoxins during toxin depuration. Part II: Blue mussels (*Mytilus edulis*) and flat oyster (*Ostrea edulis*). Toxicon.

[145] Lindegarth S., Torgersen T., Lundve B., Sandvik M. (2009). Differential retention of Okadaic Acid (OA) group toxins and Pectenotoxins (PTX) in the blue mussel, *Mytilus edulis* (L.), and european flat oyster, *Ostrea edulis* (L.). J. Shellfish Res..

[146] Jorgensen K., Scanlon S., Jensen L.B. (2005). Diarrhetic Shellfish Poisoning Toxin Esters in Danish Blue Mussels and Surf Clams. Food Addit. Contam..

[147] Suzuki T., Quilliam M.A. (2011). LC-MS/MS Analysis of Diarrhetic Shellfish Poisoning (DSP) Toxins, Okadaic Acid and Dinophysistoxin Analogues, and Other Lipophilic Toxins. Anal. Sci..

[148] Suzuki T., Mitsuya T. (2001). Comparison of dinophysistoxin-1 and esterified dinophysistoxin-1 (dinophysistoxin-3) contents in the scallop *Patinopecten yessoensis* and the mussel *Mytilus galloprovincialis*. Toxicon.

[149] Garcia C., Pruzzo M., Rodriguez-Unda M., Contreras C., Lagos N. (2010). First evidence of Okadaic acid acyl-derivative and Dinophysistoxin-3 in mussel samples collected in Chiloe Island, Southern Chile. J. Toxicol. Sci..

[150] Vale P., Sampayo M.A. (2002). Esterification of DSP toxins by Portuguese bivalves from the northwest coast determined by LC-MS-a widespread phenomenon. Toxicon.

[151] Blanco J., Moroño A., Fernández M.L. (2005). Toxic episodes in shellfish, produced by lipophilic phycotoxins: an overview. Revista Galega dos Recursos Mariños.

[152] Vale P. (2004). Differential Dynamics of Dinophysistoxins and Pectenotoxins Between Blue Mussel and Common Cockle: A Phenomenon Originating From the Complex Toxin Profile of Dinophysis acuta. Toxicon.

[153] Villar-González A., Rodríguez-Velasco M.L., Ben-Gigirey B., Botana L.M. (2007). Lipophilic toxin profile in Galicia (Spain): 2005 toxic episode. Toxicon.

[154] Suzuki T., Mackenzie L., Stirling D., Adamson J. (2001). Conversion of pectenotoxin-2 to pectenotoxin-2 seco acid in the New Zealand scallop, *Pecten novaezelandiae*. Fish. Sci..

[155] Suzuki T., MacKenzie L., Stirling D., Adamson J. (2001). Pectenotoxin-2 seco acid: A toxin converted from pectenotoxin-2 by the New Zealand Greenshell mussels, *Perna canaliculus*. Toxicon.

[156] Miles C.O., Wilkins A.L., Munday R., Dines M.H., Hawkes A.D., Briggs L.R., Sandvik M., Jensen D.J., Cooney J.M., Holland P.T. (2004). Isolation of pectenotoxin-2 from *Dinophysis acuta* and its conversion to pectenotoxin-2 seco acid, and preliminary assessment of their acute toxicities. Toxicon.

[157] MacKenzie L., Holland P., McNabb P., Beuzenberg V., Selwood A., Suzuki T. (2002). Complex toxin profiles in phytoplankton and Greenshell mussels (*Perna canaliculus*), revealed by LC–MS/MS analysis. Toxicon.

[158] Fernández M.L., Míguez A., Martínez A., Moroño A., Arévalo F., Pazos Y., Salgado C., Correa J., Blanco J., González-Gil S., Villalba A., Reguera B., Romalde J., Beiras R. (2003). First report of Pectenotoxin-2 in phytoplankton net-hauls and mussels from the Galician Rías Baixas (NW Spain) during proliferations of *Dinophysis acuta* and *Dinophysis caudata*. Molluscan Shellfish Safety.

[159] Amzil Z., Sibat M., Royer F., Masson N., Abadie E. (2007). Report on the First Detection of Pectenotoxin-2, Spirolide-A and Their Derivatives in French Shellfish. Mar. Drugs.

[160] Suzuki T., Jin T., Shirota Y., Mitsuya T., Okumura Y., Kamiyama T. (2005). Quantification of Lipophilic Toxins Associated With Diarrhetic Shellfish Poisoning in Japanese Bivalves by Liquid Chromatography-Mass Spectrometry and Comparison With Mouse Bioassay. Fish. Sci..

[161] Suzuki T., Igarashi T., Ichimi K., Watai M., Suzuki M., Ogiso E., Yasumoto T. (2005). Kinetics of Diarrhetic Shellfish Poisoning Toxins, Okadaic Acid, Dinophysistoxin-1, Pectenotoxin-6 and Yessotoxin in Scallops *Patinopecten yessoensis*. Fish. Sci..

[162] Suzuki T., Botana L.M. (2014). Lipophilic Toxins, Pectenotoxins, and Yessotoxins: Chemistry, Metabolism, and Detection. Seafood and Freshwater Toxins: Pharmacology, Physiology, and Detection.

[163] Janer G., Lavado R., Thibaut R., Porte C. (2005). Effects of 17β-estradiol exposure in the mussel *Mytilus galloprovincialis*: A possible regulating role for steroid acyltransferases. Aquat. Toxicol..

[164] Janer G., Mesia-Vela S., Porte C., Kauffman F.C. (2004). Esterification of vertebrate-type steroids in the Eastern oyster (*Crassostrea virginica*). Steroids.

[165] Wilkins A.L., Rehmann N., Torgersen T., Rundberget T., Keogh M., Petersen D., Hess P., Rise F., Miles C.O. (2006). Identification of fatty acid esters of pectenotoxin-2 seco acid in blue mussels (*Mytilus edulis*) from Ireland. J. Agric. Food Chem..

[166] Suarez-Ulloa V., Fernandez-Tajes J., Aguiar-Pulido V., Prego-Faraldo M.V., Florez-Barros F., Sexto-Iglesias A., Mendez J., Eirin-Lopez J.M. (2015). Unbiased high-throughput characterization of mussel transcriptomic responses to sublethal concentrations of the biotoxin okadaic acid. PeerJ.

[167] Martínez-Escauriaza R. (2013). Identificación de genes implicados en la eliminación de biotoxinas en el mejillón *Mytilus galloprovincialis* Lmk.: clonación y expresion de los cDNA que codifican para dos proteinas transportadoras ABC de la subfamilia B (proteínas MDR). Ph.D. Thesis.

[168] Lozano V. (2013). Identificación de genes implicados en la eliminación de biotoxinas en el mejillón *Mytilus galloprovincialis* Lmk.: clonación y expresion de los cDNA que codifican para tres proteinas transportadoras ABC pertenecientes a las subfamilias C (proteínas MRP) y G. Ph.D. Thesis.

[169] Luedeking A., Koehler A. (2004). Regulation of expression of multixenobiotic resistance (MXR) genes by environmental factors in the blue mussel *Mytilus edulis*. Aquat. Toxicol..

[170] Luedeking A., Van Noorden C.J.F., Koehler A. (2005). Identification and characterisation of a multidrug resistance-related protein mRNA in the blue mussel *Mytilus edulis*. Mar. Ecol. Prog. Ser..

[171] Eufemia N., Epel D. (2000). Induction of the multixenobiotic defense mechanism (MXR), P-glycoprotein, in the mussel *Mytilus californianus* as a general cellular response to environmental stresses. Aquat. Toxicol..

[172] Feldstein T., Nelson N., Mokady O. (2006). Cloning and expression of MDR transporters from marine bivalves, and their potential use in biomonitoring. Mar. Environ. Res..

[173] Kingtong S., Chitramvong Y., Janvilisri T. (2007). ATP-Binding Cassette Multidrug Transporters in Indian-Rock Oyster Saccostrea Forskali and Their Role in the Export of an Environmental Organic Pollutant Tributyltin. Aquat. Toxicol..

[174] Kurelec B. (1992). The multixenobiotic resistance mechanism in aquatic organisms. Crit. Rev. Toxicol..

[175] Luckenbach T., Epel D. (2008). ABCB-and ABCC-type transporters confer multixenobiotic resistance and form an environment-tissue barrier in bivalve gills. Am. J. Physiol. Regul. Integr. Comp. Physiol..

[176] Faria M., Navarro A., Luckenbach T., Piña B., Barata C. (2011). Characterization of the multixenobiotic resistance (MXR) mechanism in embryos and larvae of the zebra mussel (*Dreissena polymorpha*) and studies on its role in tolerance to single and mixture combinations of toxicants. Aquat. Toxicol..

[177] Huang L., Wang J., Chen W.C., Li H.Y., Liu J.S., Jiang T., Yang W.D. (2014). P-glycoprotein expression in *Perna viridis* after exposure to *Prorocentrum lima*, a dinoflagellate producing DSP toxins. Fish Shellfish Immunol..

[178] Blanco J., Fernández M.L., Míguez A., Moroño A. (1999). Okadaic acid depuration in the mussel *Mytilus galloprovincialis*: One- and two-compartment models and the effect of environmental conditions. Mar. Ecol. Prog. Ser..

[179] Moroño A., Fernández M.L., Franco J.M., Martínez A., Reyero I., Míguez A., Cacho E., Blanco J., Reguera B., Blanco J., Fernández M.L., Wyatt T. (1998). PSP and DSP detoxification kinetics in mussel, *Mytilus galloprovincialis*: Effect of environmental parameters and body weight. Harmful Algae.

[180] Poletti R., Viviani R., Casadei C., Lucentini L., Giannetti L., Funari E., Draisci R., Yasumoto T., Oshima Y., Fukuyo Y. (1996). Decontamination dynamics of mussels naturally contaminated with diarrhetic toxins relocated to a basin of the Adriatic Sea. Harmful and Toxic Algal Blooms.

[181] Marcaillou-Le Baut C., Bardin B., Bardouil M., Bohec M., Le Denn E., Masselin P., Truquet P., Smayda T.J., Shimizu Y. (1993). DSP depuration rates of mussels reared in a laboratory and an aquaculture pond. Toxic Phytoplankton Blooms in the Sea.

[182] Nielsen L.T., Hansen P.J., Krock B., Vismann B. (2016). Accumulation, transformation and breakdown of DSP toxins from the toxic dinoflagellate *Dinophysis acuta* in blue mussels, *Mytilus edulis*. Toxicon.

[183] Botelho M.J., Vale C., Joaquim S., Costa S.T., Soares F., Roque C., Matias D. (2018). Combined effect of temperature and nutritional regime on the elimination of the lipophilic toxin okadaic acid in the naturally contaminated wedge shell *Donax trunculus*. Chemosphere.

[184] Holmes M., Teo S., Lee F., Khoo H. (1999). Persistent low concentratrions of diarrhetic shellfish toxins in green mussels *Perna viridis* from the Johor Strait, Singapure: First record of diarrhetic shellfish toxins from South-East Asia. Mar. Ecol. Prog. Ser..

[185] Blanco J., Shumway S.E., Rodrick G.E. (2009). Modelling as a mitigation strategy for harmful algal blooms. Shellfish Safety and Quality.

[186] Svensson S. (2003). Depuration of Okadaic acid (Diarrhetic Shellfish Toxin) in mussels, *Mytilus edulis* (Linnaeus), feeding on different quantities of nontoxic algae. Aquaculture.

[187] Penry D.L. (2000). Digestive kinematics of suspension-feeding bivalves: Modeling and measuring particle-processing in the gut of *Potamocorbula amurensis*. Mar. Ecol. Prog. Ser..

[188] Pousse É., Flye-Sainte-Marie J., Alunno-Bruscia M., Hégaret H., Rannou É., Pecquerie L., Marques G.M., Thomas Y., Castrec J., Fabioux C. (2018). Modelling paralytic shellfish toxins (PST) accumulation in *Crassostrea gigas* by using Dynamic Energy Budgets (DEB). J. Sea Res..

